# 5-fluorouracil and *Rumex obtusifolius* extract combination trigger A549 cancer cell apoptosis: uncovering PI3K/Akt inhibition by in vitro and in silico approaches

**DOI:** 10.1038/s41598-024-65816-5

**Published:** 2024-06-25

**Authors:** Mikayel Ginovyan, Hayarpi Javrushyan, Svetlana Hovhannisyan, Edita Nadiryan, Gohar Sevoyan, Tigran Harutyunyan, Smbat Gevorgyan, Zaruhi Karabekian, Alina Maloyan, Nikolay Avtandilyan

**Affiliations:** 1 Research Institute of Biology, Yerevan State University, 1 Alex Manoogian, 0025 Yerevan, RA Armenia; 2 Laboratory of Immunology and Tissue Engineering, L.A. Orbeli Institute of Physiology NAS RA, Yerevan, Armenia; 3 Department of Genetics and Cytology, Yerevan State University, Yerevan, Armenia; 4 Denovo Sciences Inc, Yerevan, Armenia; 5 Center for Developmental Health, Knight Cardiovascular Institute, Oregon Health & Science University, Portland, USA

**Keywords:** Biochemistry, Cancer, Computational biology and bioinformatics, Plant sciences

## Abstract

The continuous increase in cancer rates, failure of conventional chemotherapies to control the disease, and excessive toxicity of chemotherapies clearly demand alternative approaches. Natural products contain many constituents that can act on various bodily targets to induce pharmacodynamic responses. This study aimed to explore the combined anticancer effects of *Rumex obtusifolius* (RO) extract and the chemotherapeutic agent 5-fluorouracil (5-FU) on specific molecular targets involved in cancer progression. By focusing on the PI3K/Akt signaling pathway and its related components, such as cytokines, growth factors (TNFa, VEGFa), and enzymes (Arginase, NOS, COX-2, MMP-2), this research sought to elucidate the molecular mechanisms underlying the anticancer effects of RO extract, both independently and in combination with 5-FU, in non-small lung adenocarcinoma A549 cells. The study also investigated the potential interactions of compounds identified by HPLC/MS/MS of RO on PI3K/Akt in the active site pocket through an in silico analysis. The ultimate goal was to identify potent therapeutic combinations that effectively inhibit, prevent or delay cancer development with minimal side effects. The results revealed that the combined treatment of 5-FU and RO demonstrated a significant reduction in TNFa levels, comparable to the effect observed with RO alone. RO modulated the PI3K/Akt pathway, influencing the phosphorylated and total amounts of these proteins during the combined treatment. Notably, COX-2, a key player in inflammatory processes, substantially decreased with the combination treatment. Caspase-3 activity, indicative of apoptosis, increased by 1.8 times in the combined treatment compared to separate treatments. In addition, the in silico analyses explored the binding affinities and interactions of RO's major phytochemicals with intracellular targets, revealing a high affinity for PI3K and Akt. These findings suggest that the combined treatment induces apoptosis in A549 cells by regulating the PI3K/Akt pathway.

## Introduction

The continuous increase in cancer rates, failure of conventional chemotherapies to control the disease, and excessive toxicity of chemotherapies (in some cases, including immunotherapy) clearly demand alternative approaches. Natural products contain many constituents that can act on various bodily targets to induce pharmacodynamic responses^1–5^. Modulating biochemical and immune functions using medicinal plants and their products combined with chemotherapeutic agents has recently become an accepted therapeutic approach^6^. Lung cancer is one of the most common causes of cancer-related deaths worldwide. According to the American Cancer Society, approximately 85% of all lung cancer deaths were the result of non-small-cell lung cancer (NSCLC).

Targeting the PI3K/Akt signaling pathway and related extracellular and intracellular components is an essential goal for several reasons. Correctly regulating the activity and quantity of these components can have a strong anticancer effect on cancer cells. Several pro-tumorigenic processes converge on hyperactive PI3K/Akt signaling, and it is becoming increasingly evident that reactive oxygen species (ROS) metabolism is no exception to this^7^. Metabolic reconfiguration and the resulting generation of ROS are vital for facilitating the development of tumors. Dysregulated PI3K/Akt signaling is crucial in regulating numerous molecular processes that elevate ROS levels. This occurs by directly influencing mitochondrial energy production and activating NADPH oxidases (NOXs) or indirectly generating ROS as a metabolic byproduct^8^. Comprehending the intricate relationship between ROS and PI3K/Akt signaling is vital for devising effective therapeutic approaches to combat tumors reliant on this pathway. This significance is underscored by recent clinical trials showing limited efficacy of PI3K/Akt pathway inhibitors and the emergence of resistance. These findings could lead to the discovery of new biomarkers and metabolic vulnerabilities, as well as the development of more potent therapeutic combinations that disrupt redox balance and specifically target PI3K-driven tumors^8,9^. Chemotherapy and radiotherapy stand as primary treatments for cancer patients, and they induce apoptosis in cancer cells partly by increasing ROS levels. Agents like 5-FU, cisplatin, and other chemotherapeutic drugs trigger ROS production by influencing the electron transport chain. The heightened ROS levels then trigger cascades, such as caspase activation, release of cytochrome C, and DNA damage, ultimately leading to apoptosis^8^. In this study, we incorporated a chemotherapeutic compound alongside 5-FU. This approach allows for the concurrent inhibition of the PI3K/Akt pathway while maintaining or increasing significant levels of ROS and reactive nitrogen species (RNS). Such elevation effectively promotes the induction of apoptosis.

The PI3K pathway stimulates metastasis by promoting tumor neovascularization, which is required for the metastatic spread of tumors. PI3K forms a complex with E-cadherin, β-catenin, and VEGFR-2 and is involved in endothelial signaling mediated by VEGF by activating the PI3K/Akt pathway^10^. The PI3K/Akt signaling pathway also promotes TNF-induced endothelial cell migration and regulates tumor angiogenesis. TNF-α plays a significant role in promoting the survival and metastasis of lung cancer. The levels of TNF-α in tumor tissues and serum collected from patients with NSCLC substantially increase with the clinical stage of the tumor. Additionally, matrix metalloproteinases (MMPs) and cyclooxygenase-2 (COX-2) contribute to tumor angiogenesis. COX-2 stimulates endothelial angiogenesis primarily through upregulating the antiapoptotic protein Bcl-2 and activating the PI3K/Akt signaling pathway^7,11–13^.

The literature reveals that TNF-α, VEGF-α, COX-2, MMP-2, Caspase-3, and NOS are interconnected in various cancer processes. Their interrelations are mediated through the PI3K/Akt signaling pathway. This study focused on employing a combination of natural compounds and a chemotherapeutic agent to target these specific components to attain potent anticancer effects.

We hypothesized that phytoextracts, either single or in combination with chemotherapy compounds, may effectively modulate the immune system (TNFa/COX-2/Arginase), inhibit angiogenesis and progression of metastasis (VEGFa/NOS/NO/MMP-2) via regulation of PI3K/Akt signaling pathway. Our previous research showed the promising anticancer effect of *Rumex obtusifolius* (RO) in an in vivo experimental breast cancer rat model; in parallel, its cytotoxic effect was elucidated against two cell cultures: MCF-7 and HT29^14^. The combined anticancer effects of inhibitors targeting the metabolic pathway of L-arginine were also investigated. The findings demonstrate significant anti-tumor properties, including tumor size, number, and mortality reductions. Changes in various blood biochemical parameters associated with L-arginine metabolic participants were observed. However, alterations in key factors that would provide a detailed understanding of the exact molecular mechanisms underlying the anticancer effects were not noted^15^. The current study elucidated the mechanisms of the anticancer effects of the *R. obtusifolius* extract, both independently and in combination with the conventional chemotherapeutic compound 5-FU. Specifically, the impact of the herbal extract of *R. obtusifolius* (0.25 mg/mL) on the TNFα-VEGFα/PI3K/Akt/NOS/COX-2-MMP-2 pathway was assessed separately and in combination with 5-FU (40 µM) in non-small lung adenocarcinoma A549 cells. In addition, the possible interaction of compounds identified by HPLC/MS/MS of the effect of RO on PI3K/Akt in the active site pocket was also elucidated by an in silico study. Uncovering the molecular mechanisms of their anticancer effects, identifying the most active phytochemicals, and clarifying the specific targets of these compounds create a potential to inhibit, prevent, or delay cancer development with minimal side effects.

## Results

### Assessment of the growth-inhibiting properties of 5-fluorouracil with *R. obtusifolius* extract by an MTT assay

**Figure 1:**
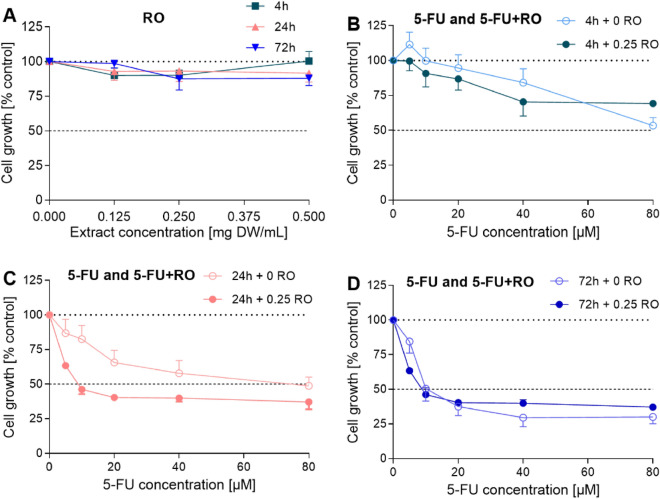
Growth rate of A549 cells treated with the RO extract for 4, 24, and 72 h (**A**). The growth-inhibiting effect of 5-fluorouracil separately and in combination with the none-inhibitory concentration of RO extract (0.25 mg DW/mL) on A549 cells at 4 (**B**), 24 (**C**), and 72 h (**D**). The results represent the means ± SD from three independent experiments; SD values did not exceed 15%.

The growth-inhibiting properties of the RO seed ethanol extract on A549 cancer cells were evaluated using an in vitro MTT assay. The RO extract, even at the highest tested concentration (0.5 mg DW/mL) and at any of the tested exposure times (4, 24, and 72 h), did not show any statistically significant impact on the growth of the A549 cells (Fig. 1A).

Further modulating activity of the non-inhibitory concentrations of the RO extract (0.25 mg DW/mL) with fluorouracil (5-FU) on A549 cells (Fig. 1B–D) was investigated using in vitro MTT assay. There was a statistically significant strong modulation with 5-FU at 24 h exposure time with all the tested 5-FU concentrations (Fig. 1C). Considerable modulation was also detected at 4 h of exposure time (Fig. 1B). However, at 72 h, no modulation was observed (Fig. 1D).

### *R. obtusifolius* extract alone and in combination with 5-FU downregulates TNFa, VEGFa, COX-2, and MMP-2.

**Figure 2:**
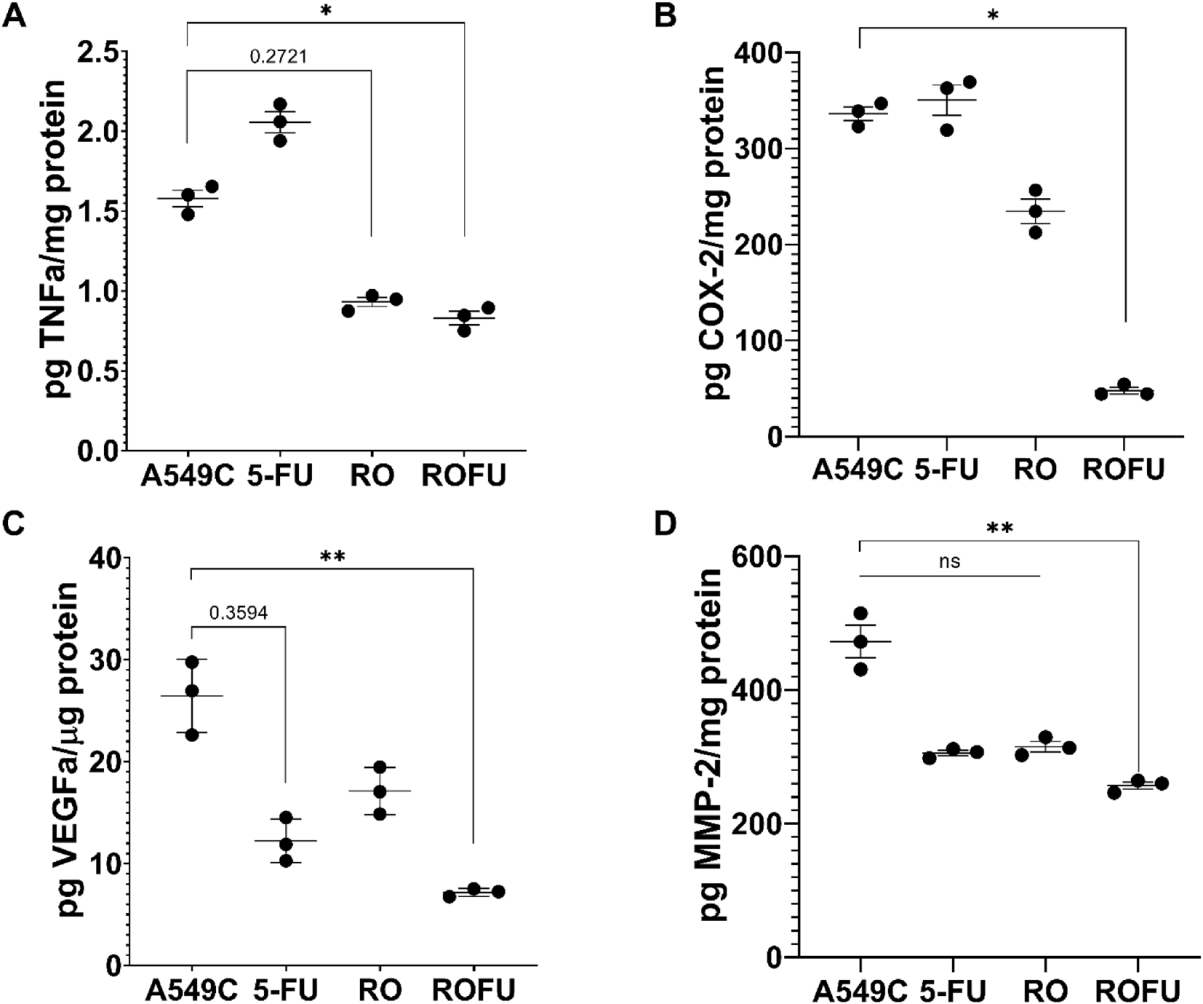
The influence of the *R. obtusifolius* extract alone and in combination with 5-FU on TNFa (**A**), COX-2 (**B**), VEGFa (**C**), and MMP-2 (**D**) in A549 cells. Control—A549C, 5-Fluorouracil—5-FU (40 μM), *Rumex obtusifolius*—RO (0.25 mg/mL), ROFU—RO + 5-FU (0.25 mg/mL + 40 μM). Each test sample was added to three different passages in triplicate (n = 3, *—*p* ≤ 0.05, **—*p* ≤ 0.01, ns—non-significant).

Using ELISA, changes in TNFα-related COX2 and VEGF-related MMP2 were assessed. The RO extract decreased TNFα (Fig. 2A) and VEGFα (Fig. 2B) in the cell medium by 40% and 33%, respectively, and the quantities of COX-2 and MMP2 by 31.5% and 33%, respectively.

5-FU alone does not affect TNFa/COX-2, but when combined with the plant extract, the effect was greater than with the plant extract alone or 5-FU alone (*p* ≤ 0.05). The most visible modulatory effects were TNFα (the combined effect decreased it close to the value of RO), VEGFa (33% and 50% decrease compared to 5-FU and RO alone, respectively, *p* ≤ 0.01), and COX-2 (the reduction was about 90% for 5-FU and 80% for RO). In the case of COX-2, a synergistic phenomenon was documented.

### Regulation of PI3K/Akt pathway

The downstream signaling pathway was elucidated to understand further the cause of decreased TNFα, VEGFa, COX-2, and MMP-2 levels and their effect on the cell.

**Figure 3:**
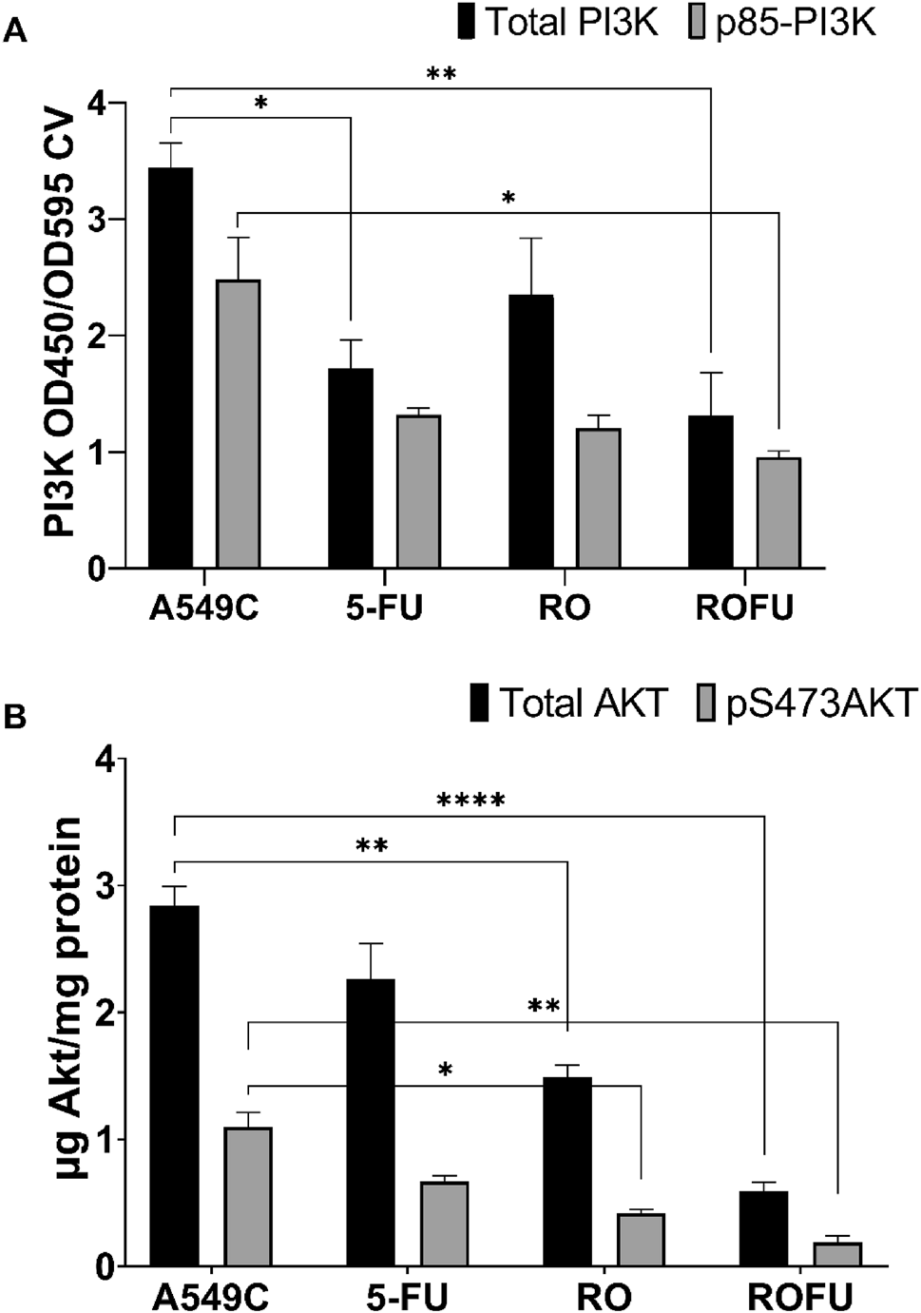
Effect of RO, 5-FU, and their combination on the PI3K/Akt pathway in A549 cells (A-PI3K, B—Akt). Total and phospho-kinases were assayed in triplicate using the phospho- and total kinase antibodies included in the PI 3 Kinase and Akt kits (n = 3, *—*p* ≤ 0.05, **—*p* ≤ 0.01, ***—*p* ≤ 0.001, ****—*p* ≤ 0.0001). p85-PI3K—Phospho-PI 3 kinase p85, pS473AKT—phospho-Akt (Ser473).

RO acts as an inhibitor of the Pi3K/Akt pathway. In particular, total and phosphorylated PI3K and Akt were reduced after treating the A549 cells with the RO extract (Fig. 3A). The combination of RO and 5-FU reduced the amount of total and phosphorylated PI3K by about 2.5-fold compared to the control cells (*p* ≤ 0.01). RO alone did not affect these two forms of PI3K, and 5-FU only affected the total amount of PI3K. RO and 5-FU reduced both the phosphorylated and total Akt levels, and the combination increased this reduction several-fold showing a synergistic effect (Fig. 3B). We assumed that RO works through the TNFa-PI3K-Akt cascade. Thus, changes in the amount and activity of NO, MDA, Arginase, and NOS participants were elucidated to understand the mechanism further.

### Stimulation of RNS and ROS by regulating arginase and NOS activity

**Figure 4:**
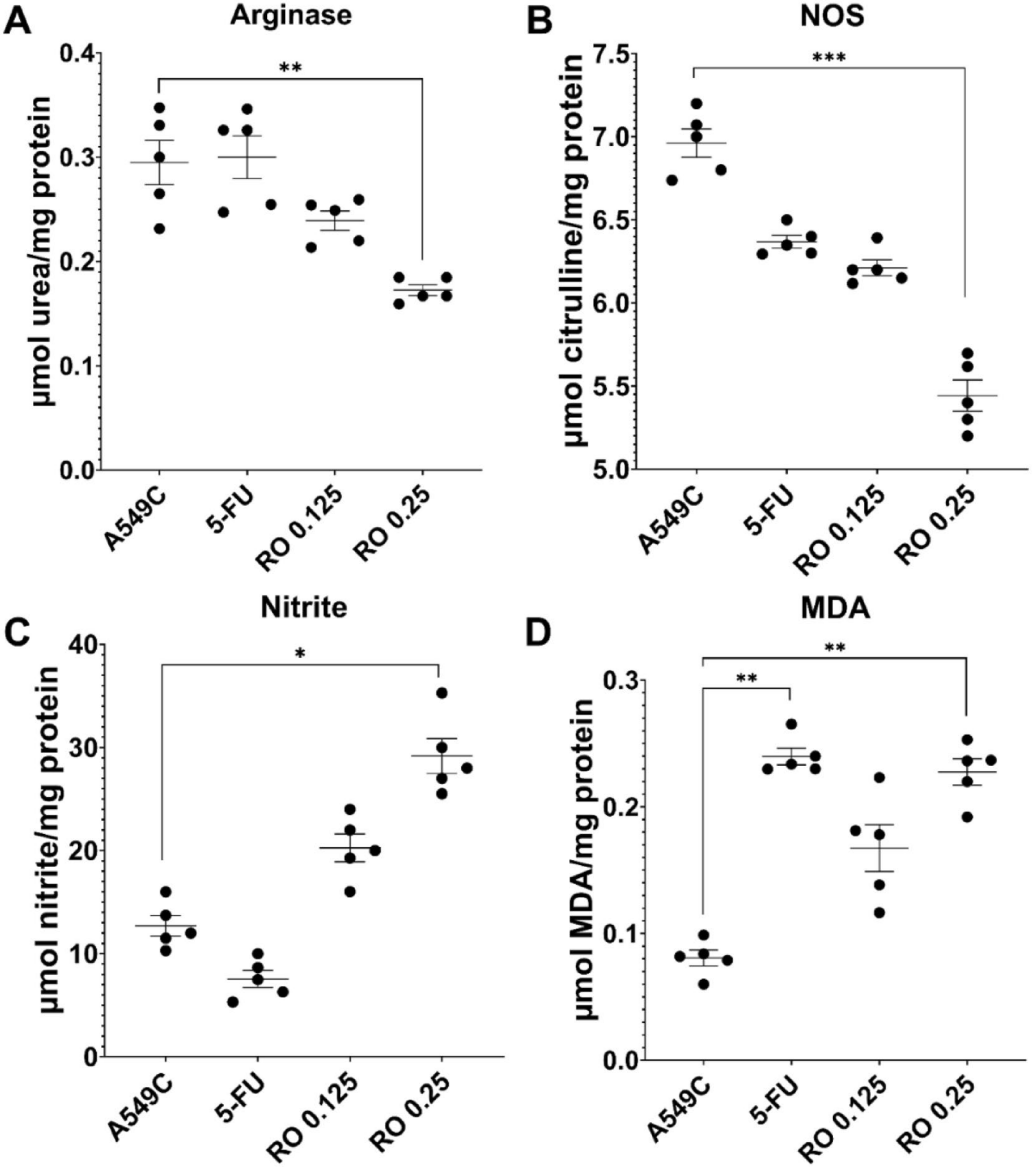
Stimulation of RNS and ROS by RO regulates arginase (**A**) and NOS (**B**) activity, nitrite anions (**C**), and MDA (**D**) quantity in A549 cells. Each condition was added to five different passages in triplicate (n = 5, *—*p* ≤ 0.05, **—*p* ≤ 0.01, ***—*p* ≤ 0.001, ****—*p* ≤ 0.0001).

Since, in most cases, COX-2 interacts with arginase and NOS^16^, changing the concentration of VEGFa with NO and NOS and TNFa with ROS and RNS was valuable and necessary to observe the activities of arginase, NOS, and quantitative changes of NO and MDA (Fig. 4).

By reducing VEGF, the plant extract also inhibited NOS activity (*p* ≤ 0.001) but increased the quantity of NO (*p* ≤ 0.05, Fig. 4B,C). Since the activity of NOS was depressed, but the amount of nitrite ions increased, the increase in RNS may be due to the increase in ROS. ROS and generated NO most likely lead to RNS generation and ROS/NO-mediated apoptosis. The latter was confirmed by the high quantity of MDA, which was promoted by the plant (*p* ≤ 0.01, Fig. 4D). Typically, in cancer, activation of the PI3K/Akt pathway leads to increased ROS^17,18^; conversely, increased ROS leads to activation of this pathway. However, this phenomenon was not observed in our study. In this case, the plant adjusted so that suppressing the PI3K/Akt pathway increased the amount of ROS and RNS. This indicated a multi-target effect of the compounds contained in the herb. To verify that increased RNS and ROS quantities, as well as inhibition of the PI3K/Akt pathway, leads to apoptosis, Caspase-3 activity was assessed, and chromatin staining was performed with Hoechst 33258 dye to observe segmentation and condensation.

### Assessment of apoptosis by Hoechst 33258 staining

**Figure 5:**
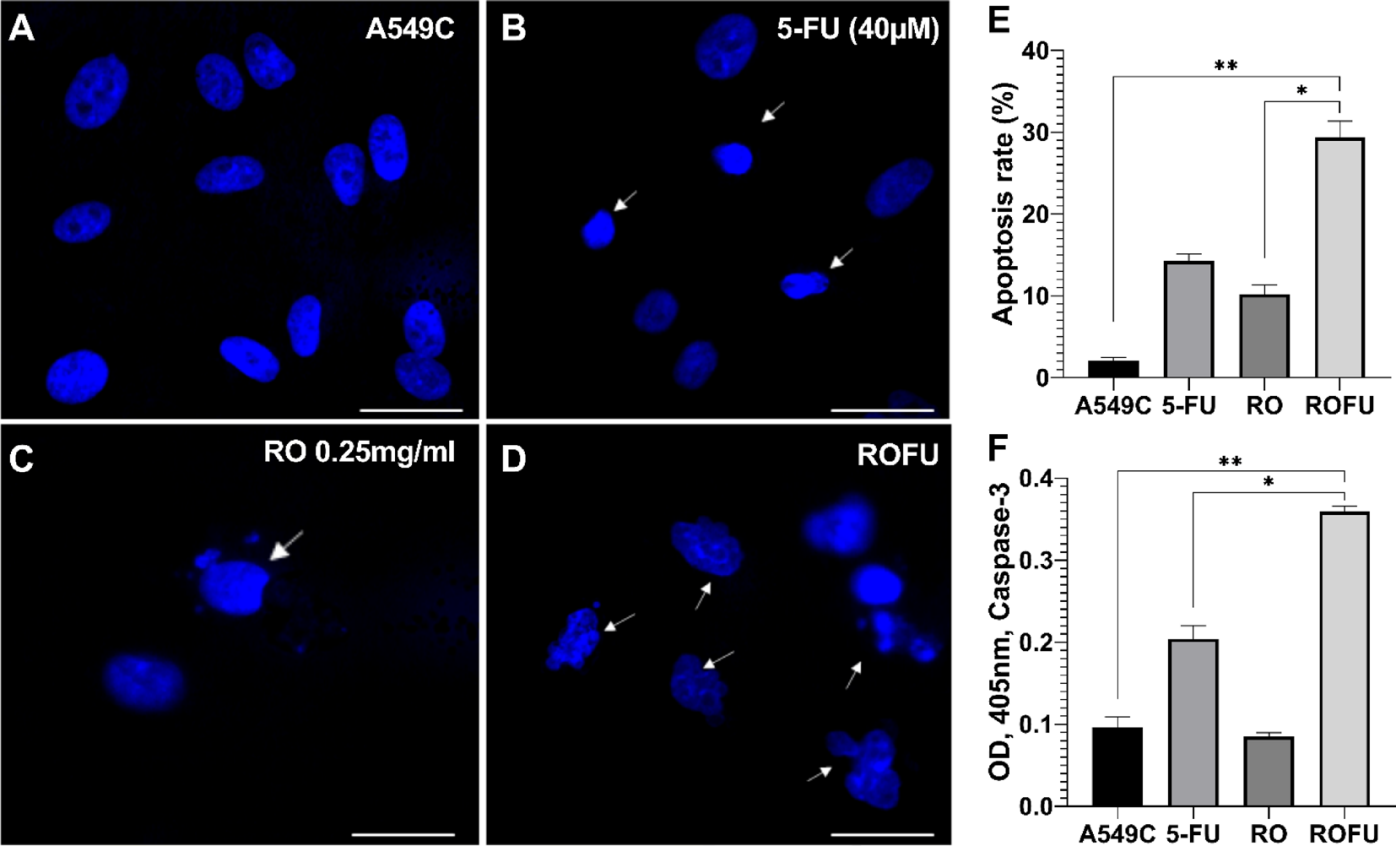
Hoechst 33258 staining (blue) assay to assess apoptosis in A549 cells. Apoptotic cells are indicated with white arrows. The scale bar is 100 μm. (**A**) Untreated cells (A549C) have smooth edges and dispersed fluorescence (**B**,**E**). 5-FU-induced apoptosis can be seen by the occurrence of pyknotic cells with condensed chromatin (**C**,**E**). Incubation with RO elevated the number of apoptotic cells with rough edges of nuclei (**D**,**E**). The combined treatment of cells with 5-FU + RO resulted in cells with signs of nuclei fragmentation and chromatin condensation. (**E**) Apoptosis rate evaluated by the Hoechst 33258 staining, *p < 0.05—compared with the RO, **p < 0.01—compared with the control. (**F**)—caspase-3 activity evaluated by the colorimetric assay, *p < 0.05—compared with the 5-FU, **p < 0.01—compared with the control.

Hoechst 33258 staining allows discrimination of apoptotic and non-apoptotic cells based on morphological changes of the nuclei (Fig. 5). The nuclei in normal cells exhibited evenly dispersed and weak fluorescence and smooth edges (Fig. 5A). Apoptotic cells were distinguished by condensed chromatin (Fig․ 5B, marked with arrows) and the rough edges of their nuclei (Fig. 5C, marked with arrows), as well as signs of nuclear fragmentation (Fig. 5D, marked with arrow). The results indicated that incubating A549 cells with 5-FU, RO, and their combination for 24 h significantly increased the rate of apoptotic cells (Fig․ 5E). In the control cells, the apoptosis rate was 2.40 ± 0.56%. Treatment with 5-FU or RO alone significantly increased the rate of apoptotic cells up to 14.70 ± 1.83% and 11.30 ± 0.98%, respectively. At the same time, the combination of 5-FU + RO elevated the rate of apoptotic cells up to 29.5 ± 4.94% (*p* < 0.01). Thus, the apoptosis of A549 cells induced by the combination of 5-FU + RO was significantly higher when compared with that of 5-FU or RO alone (*p* < 0.05).

Since Caspase-3 is active in the execution phase of apoptosis, we next used a colorimetric assay to determine whether it was activated following treatment with 5-FU, RO, and their combination 5-FU + RO for 24 h (Fig․ 5F). The spectrophotometric analysis revealed a significant increase in caspase-3 activity in all treatment variants, which was more pronounced in the cells treated with 5-FU + RO (*p* < 0.01).

### The effect of the interaction of potential compounds on PI3K and Akt docking

**Figure 6:**
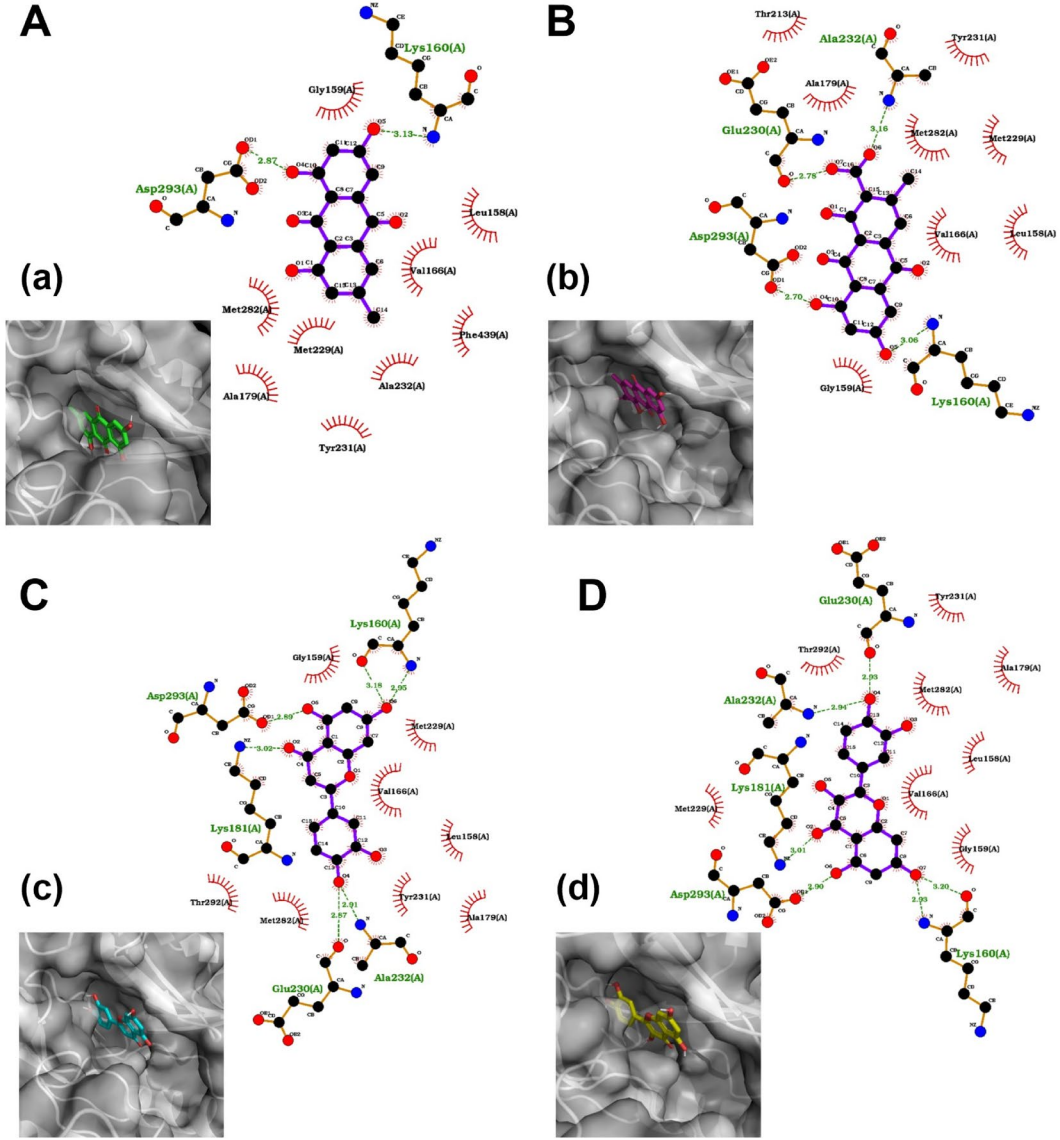
2D binding analysis and interaction types for AKT in combination with 3D visualization. 3D Visualizations: (**A**): Emodin interaction diagram. Panel (**a**) shows the surface representation of the binding pocket with Emodin in green sticks. (**B**): Endocrocin interaction diagram. Panel (b) displays the surface representation of the binding pocket with Endocrocin in magenta sticks. (**C**) Luteolin interaction diagram. Panel (c) presents the surface representation of the binding pocket with Luteolin in cyan sticks. (**D**) Quercetin interaction diagram. Panel (d) illustrates the surface representation of the binding pocket with Quercetin in yellow sticks. In each 2D interaction diagram, the atoms are colored as follows: carbon (black), oxygen (red), nitrogen (blue), and hydrogen (not shown for clarity). Amino acids forming hydrogen bonds with the ligands are labeled, and their interactions are shown with green dotted lines and the bond distances in angstroms. Hydrophobic interactions are represented by red semicircles around the interacting amino acids. Amino acids involved in hydrophobic interactions are labeled in red, and those involved in hydrogen bonding are labeled in green.

**Figure 7:**
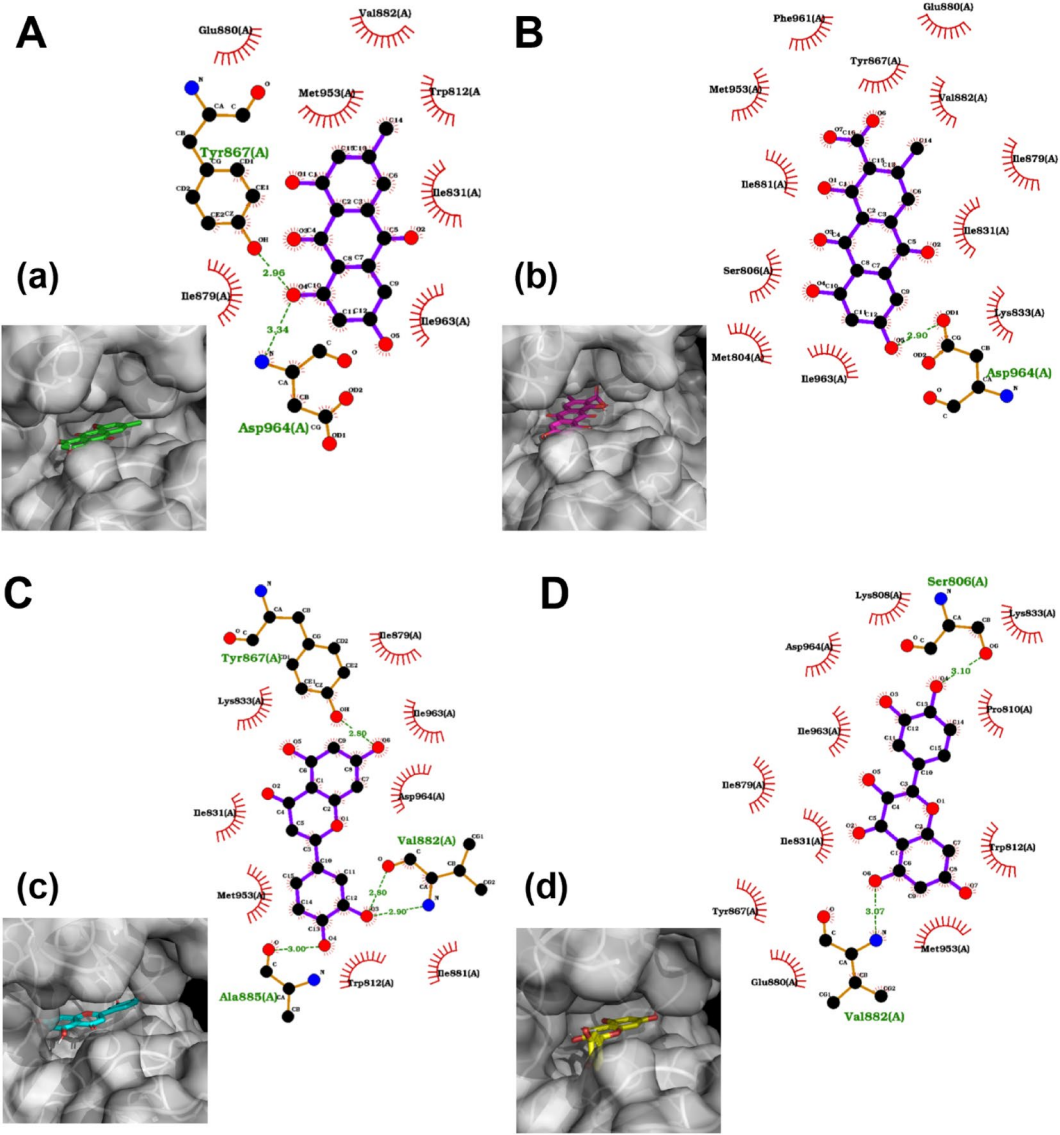
2D binding analysis and interaction types for PI3K in combination with 3D visualization. 3D Visualizations: (**A**): Emodin interaction diagram. Panel (a) shows the surface representation of the binding pocket with Emodin in green sticks. (**B**): Endocrocin interaction diagram. Panel (b) displays the surface representation of the binding pocket with Endocrocin in magenta sticks. (**C**): Luteolin interaction diagram. Panel (c) presents the surface representation of the binding pocket with Luteolin in cyan sticks. (**D**): Quercetin interaction diagram. Panel (d) illustrates the surface representation of the binding pocket with Quercetin in yellow sticks. In each 2D interaction diagram, the atoms are colored as follows: carbon (black), oxygen (red), nitrogen (blue), and hydrogen (not shown for clarity). Amino acids forming hydrogen bonds with the ligands are labeled, and their interactions are shown with green dotted lines and the bond distances in angstroms. Hydrophobic interactions are represented by red semicircles around the interacting amino acids. Amino acids involved in hydrophobic interactions are labeled in red, and those involved in hydrogen bonding are labeled in green.

The docking of the top compounds present in the RO ethanolic extract (Suppl. Table) by Autodock Vina was performed on the binding pockets of AKT (PDB ID: 2JDO) and PI3K (PDB ID: 6 AUD) (Figs. 6, 7). Each crystallographic structure’s binding pocket contained a bound ligand, which was extracted and rocked as a control. The docking results are presented in Table 1. Based on the average score of the two target proteins, none of the compounds showed a better docking score (the lower, the better) than the redocking score of the PI3K control. However, 14 out of 17 investigated compounds demonstrated better docking scores than the redocking score of the AKT control ligand. Therefore, the extracted compounds of interest may have a better affinity toward AKT.

**Table 1:** Docking results of RO on PI3K and Akt.

Compound	AKT (2JDO)	PI3K (6AUD)	Average
PI3K-control ligand		− 9.6	− 9.6
Endocrocin	− 9.1	− 9.2	− 9.15
Emodin	− 8.8	− 9.2	− 9
Luteolin	− 8.5	− 8.8	− 8.65
Quertecin	− 8.5	− 8.4	− 8.45
Epicatechin-gallate	− 8.5	− 8.3	− 8.4
Eriodictol	− 8.3	− 8.5	− 8.4
Quercetin-3-D-galactoside	− 8.3	− 8.5	− 8.4
Hamamelofuranose	− 8.4	− 8.2	− 8.3
Isorhamnetin-3-O-glucoside	− 8	− 8.5	− 8.25
Catechin	− 7.8	− 8.5	− 8.15
Epicatechin	− 8.1	− 7.8	− 7.95
Apigenin-sulfate	− 8.1	− 7.6	− 7.85
Qurecetin-diglucoside	− 7.5	− 7.8	− 7.65
4-glucogallic acid	− 7.5	− 6.9	− 7.2
AKT-control ligand	− 7		− 7
Procyanidin-dimer	− 7.1	− 6.3	− 6.7
Protocatechuic-acid	− 5.7	− 5.6	− 5.65
Hydroxybenzoic-acid	− 5.5	− 5.5	− 5.5

Understanding the absorption, distribution, metabolism, and excretion (ADME) properties of drug candidates is essential due to their profound influence on therapeutic agents' pharmacokinetics, efficacy, and potential side effects^19^. For this reason, we computationally analyzed the ADME characteristics of the 17 leading compounds using the SwissADME online service, and the key features are outlined in Table 2. A variety of physical and chemical properties, such as molecular weight (MW), partition coefficient (LogP), hydrogen bond acceptors (HBA), and hydrogen bond donors (HBD), were assessed. Based on these calculations, we examined how the compounds adhere to Lipinski’s rule of 5, which is a set of rule-of-thumb guidelines in medicinal chemistry that predict oral bioavailability in drugs, stating that a molecule will likely be an effective oral medication when it does not violate more than one of these rules: no more than 5 hydrogen bond donors, 10 hydrogen bond acceptors, a molecular mass less than 500 Daltons, and a LogP not exceeding 5^20^. The results illustrate that 10 of the 17 compounds did not violate Lipinski’s rule of 5, indicating their potential to be drug candidates. Remarkably, these 10 compounds included the top 4 with the best docking scores (namely, endocrocin, emodin, luteolin, and quercetin).

**Table 2:** ADME properties of the RO extract phytochemicals.

Compounds	MW	HBA	HBD	LogP	Num of Viol
Endocrocin	314	7	4	1.43	0
Emodin	270	5	3	1.87	0
Luteolin	284	6	4	1.73	0
Quercetin	299	7	5	0.17	0
Epicatechin gallate	442	10	7	1.25	1
Erodictol	280	6	4	0.84	0
Quercetin 3-D-galactoside	464	12	8	− 0.25	2
Hamamelofuranose	180	6	5	− 1.94	0
Isorhamnetin 3-O-glucoside	478	12	7	− 0.15	2
Catechin	290	6	5	0.85	0
Epicatechin	290	6	5	0.85	0
Apigenin sulfate	364	9	3	1.28	0
Qurecetin diglucoside	607	17	11	− 2.8	3
4 glucogallic acid	332	10	7	1.9	1
Procyanidin dimer	562	12	10	0.54	3
Protocatechuic acid	151	4	3	0.4	0
Hydroxybenzoic acid	134	3	2	0.72	0

Accordingly, we decided to examine further the 4 compounds mentioned above. In particular, we analyzed the binding mode of their best docking scores for the two proteins in addition to a 3D visual inspection (Figs. 6, 7). Based on the analysis, emodin formed hydrogen bonds with Asp293 and Lys160 amino acids of AKT. The interactions with 9 other amino acids were hydrophobic (Fig. 6A). With PI3K, emodin formed hydrogen bonds with Tyr867 and Asp 964. The interactions of emodin and Glu880, Val882, Trp812, Ile831, Ile963, and Ile879 interactions were hydrophobic (Fig. 7A). Regarding endocrocin and AKT interactions, there were hydrogen bonds with Glu230, Ala232, Lys160, and Asp293. In addition, there were hydrophobic interactions with 8 other amino acids (Fig. 6B). Endocrocin formed only one hydrogen bond, namely with Asp963, with PI3K. Furthermore, there were hydrophobic interactions with 12 additional amino acids (Fig. 7B).

Luteolin formed hydrogen bonds with the Lys181 and Asp293 amino acids of AKT, complemented by 8 other hydrophobic interactions (Fig. 6C). Regarding PI3K, luteolin formed one hydrogen bond with Tyr867 and Ala885 and two hydrogen bonds with Val882. Furthermore, there were hydrophobic interactions with 8 other amino acids (Fig. 7C). Finally, Quercetin formed four hydrogen bonds with Asp293, Lys181, Ala232, and Glu230 of AKT. In addition, there are 8 hydrophobic interactions (Fig. 6D). Regarding PI3K, quercetin formed hydrogen bonds with Ser806 and Val882. This was complemented by hydrophobic interactions with 10 other amino acids (Fig. 7D). The specific interactions between emodin, endocrocin, luteolin, and quercetin with the target proteins AKT and PI3K highlight their potential to modulate the function of these proteins. Notably, while all four compounds formed hydrogen bonds with key residues in AKT, the variety and number of interactions differed, which may influence their affinity and specificity. The prevalence of the hydrophobic interactions alongside hydrogen bonds, particularly with PI3K, underscores the potential of these compounds to anchor firmly within the binding pockets, possibly conferring stable interactions and effective inhibition. Such differential binding patterns could translate into varying degrees of therapeutic efficacy and selectivity among these compounds.

## Discussion

Although chemotherapy is the most commonly used treatment, it also kills normal cells, causing many side effects. Therefore, it is urgent to develop novel alternative therapeutic strategies to overcome these problems. Many phytochemicals have been isolated from various plants that have regulatory effects on the targets considered in our study. We hypothesized that the RO extract, either alone or in combination with chemotherapy compounds, may effectively modulate the immune system (TNFa/COX-2/Arginase), inhibit angiogenesis and progression of metastasis (VEGFa/NOS/NO/MMP-2) via regulation of the PI3K/Akt signaling pathway.

The study examined the effect of RO on 5-FU-induced apoptosis in A549 cells. An MTT assay showed that RO alone did not induce noticeable inhibition of the growth of A549 cells. This is interesting as, according to our previous research, the RO seed extract expressed strong cytotoxic activity on two tested cancer cell lines (HT29 and MCF-7) at even 0.125 mg DW/mL concentration^14^. Although the RO extract did not inhibit the growth of A549 cells, we assumed that acting synergically would increase the cytotoxic properties of chemotherapeutic agents like Fluorouracil. The speculations were made based on earlier studies, where the RO extract, when combined with NG-nitro-L-arginine methyl ester (NOS inhibitor) and NG-hydroxy-nor-L-arginine (arginase inhibitor), increased their therapeutic effects probably by regulating redox homeostasis^14^. The experiments were done in an in *vivo* rat mammary carcinogenesis model, and based on the obtained data, the RO extract possessed a modulating effect on 5-FU. It is essential to point out that RO had a synergic effect rather than an additive, as the RO extract did not show any growth-inhibiting effect at the concentration used in the combined treatment. The modulating properties of the RO seed extract could have great importance, considering that modulation of the anticancer effects of chemotherapy drugs through plant extracts or derived compounds is a promising strategy to overcome drug resistance and reduce side effects^21^.

Different biochemical parameters were explored to understand further biochemical mechanisms underlying modulating properties RO on 5-FU, including quantitative changes of TNFa, VEGFa, COX-2, and MMP-2, regulation of PI3K/Akt pathway, assessment of apoptosis, etc*.* We considered the PI3K/Akt signaling pathway because it is a major signaling pathway in various types of cancer. It controls the hallmarks of cancer, including cell survival, angiogenesis, inflammation, metastasis, and metabolism. According to the literature, the VEGF is the most potent stimulant of angiogenesis and activates NOX isoforms directly or indirectly through PI3K/Akt induction^8^. After activation by VEGF, Akt promotes the proliferation, migration, and survival of endothelial cells, thus affecting angiogenesis. This finding also supports the conclusion that endothelial nitric oxide synthase (eNOS), which controls vascular tone, is a specific substrate of Akt1 in endothelial cells^7^. The subsequent production of superoxide and hydrogen peroxide is necessary to regulate transcription factors, which promote angiogenesis, including NF-κB, MMPs, COX-2, and HIF-1α. COX-2 is up-regulated in many malignant cancers, including gastric, colon, breast, esophagus, pancreas, hepatocellular carcinoma, and NSCLC. The overexpression of COX-2 effectively potentiates the cisplatin and other chemotherapy drug resistance of NSCLC cells by promoting EMT^22^.

Our data showed that RO extracts significantly decreased the quantities of TNFα, VEGFa, COX-2, and MMP2 in A549 cancer cells in combination with 5-FU. Inflammatory cytokines, growth factors, and their receptors, such as TNF, TNFR, VEGF, and VEGFR, act as positive regulators to transmit signals to mTOR through the PI3K/Akt pathway^7^. These factors also play an important role in various immune system regulation processes. Researchers found that neoadjuvant immunotherapy for NSCLC, immune checkpoint inhibitors for melanoma, and adjuvant immunotherapy for melanoma and hepatocellular carcinoma are extremely relevant but still underdeveloped directions for this field warrant further investigation^22^. Models like the one we propose (herb + chemotherapeutic compound) can be further incorporated into any neoadjuvant and adjuvant immunotherapy phases. PI3K/Akt signaling blocks the expression of proapoptotic proteins, reduces tissue apoptosis, and increases the survival rate of cancer cells^7^. Akt inhibits the proapoptotic factors Bad and procaspase-9 through phosphorylation and induces the expression of the proapoptotic factor Fas ligand. In addition, Akt activation is associated with resistance to increased apoptosis induced by TNF. During routine chemotherapy, no treatment interval exists, allowing resistant cells to be generated and leading to tumor regeneration. The PI3K/Akt signaling pathway is important for the drug resistance of different types of cancer, such as lung cancer and esophageal cancer. For NSCLC cells with high Akt expression, PI3K/Akt signaling pathway inhibitors increase chemotherapy-induced apoptosis and reduce their resistance to chemotherapy^23^. Therefore, inhibition of the PI3K/Akt signaling pathway, which has been shown to regulate cancer cell apoptosis can serve as a new direction for future research on cancer treatment^7^. This work is important because the obtained results touch on the question of plant pro-oxidation. Increased malondialdehyde and nitrite ions are present in the cellular environment, indicating increased ROS and RNS. According to the literature, the latter is also regulated by Akt^8^. In addition, the change in Akt activity also affects the regulation of Caspase-3 activity and, therefore, apoptosis. Given that cell leakage may be a factor in RNS- and ROS-mediated apoptosis, the alteration of Caspase-3 activity was observed. The possibility of chromatin segmentation and condensation under the effect of herb and combination was also studied by Hoechst stain to elucidate the stimulation of apoptosis further. Hoechst staining revealed an increase in the rate of apoptotic cells after treatment with 5-FU (40 uM) or RO alone. The combination of 5-FU + RO synergistically evoked Caspase-3 activity; thus, RO elevated the frequency of 5-FU-induced apoptosis. The results obtained in the case of combinations of herbs and chemotherapeutic agents showed a decrease in TNFa and VEGFa and an increase in NO and MDA quantity. The latter indicated ROS/RNS-mediated cytotoxicity of herbs in the tumor microenvironment. Many factors can damage DNA, proteins, or lipids in cells directly or indirectly, such as exogenous drugs, endogenous reactive oxygen species, or free radicals. During this process, the transcription factor nuclear factor erythroid 2-related factor 2 (Nrf2) is considered a significant modulator, maintaining the cellular redox balance by expressing antioxidant proteins^24,25^. Several cancer chemopreventive compounds targeting Nrf2 have been reported, such as Oltipraz, Sulforaphane, Curcumin, Resveratrol, and Luteolin^24^. The increase in MDA and NO amount of RO + 5-FU combination is possibly promoted by the modulation of the Nrf2 pathway itself, which will be clarified in future studies.

Decreased COX-2, Arginase, and MMP-2 were observed in the A549 cells under the influence of the herb extracts and combinations. The work is also highlighted by considering the herb and the classical chemotherapeutic compound 5-fluorouracil, which has a broad spectrum effect and is used in chemotherapeutic cocktails^26^. It was important to observe the herb-drug interaction and identify whether there was a synergistic effect between this herb and 5-FU. Even though several studies show the anticancer effect of various herbs, and our in vivo model showed the effective use of this herb against breast cancer in combination with L-arginine metabolic pathway inhibitors, few studies have revealed the mechanisms by which this effect occurs. This work is also valuable in that, by using a multi-component decoction of the medicinal plant, the possible protection of these compounds against PI3K and Akt enzymes was also clarified by a parallel in silico study. There are 3 main findings. Elucidated the mechanisms of the anticancer effect of an unexplored herb by looking at the TNFa/PI3K/Akt/COX-2/ARG/NOS/ROS/RNS/Caspase-3 pathway demonstrated an herb-drug synergistic interaction affected by different compounds, which were revealed based on in silico studies. These compounds also had the greatest affinity for PI3K/Akt, which may play a key role in RO extracts with promising anticancer properties. In our previous work in another cell culture (MCF-7), RO has also been shown to have a down-regulating effect on total and phosphorylated amounts of PI3K^27^. Another important finding of the work is that the quantitative data of the MDA and nitrite anions differed from our previous studies in vivo ^14^. During earlier in vivo studies on the rat mammary carcinogenesis model, a decrease in the amount of malondialdehyde and nitrite ions was observed in the blood. At the same time, an increment of their quantity was detected in the cell culture. The circumstance of selective effect is also seen here, thanks to which it is possible to deliver these active compounds to the tumor environment through delivery systems and to leave a point effect on the targets presented^28,29^. Then, we elucidated the main compounds of the RO extract that might have promising anticancer properties. In our previous research, more than 200 phytochemicals were identified in the ethanol extract of RO ethanol extract based on LC-Q-Orbitrap-HRMS analysis. The full list of identified compounds in RO ethanol extract is presented in earlier work^14^. During this study, the in silico analyses revealed that 4 of these compounds (namely, endocrocin, emodin, luteolin, and quercetin) had a high affinity for PI3K and Akt, indicating that the downregulation of the PI3K/Akt pathway by the herbs may be responsible for their beneficial effects on the quantitative changes in the explored factors and enzymes. The results demonstrated that all 4 compounds formed at least 2 hydrogen bonds and at least 6 hydrophobic interactions with amino acids of the binding pockets of both AKT and PI3K. The only exception was the endocrine-PI3K interaction, with only one hydrogen bond. Nevertheless, this was amply compensated with an additional 12 hydrophobic interactions. The analysis indicated strong interactions in the case of all 8 ligand–protein pairs, which can potentially change both proteins’ function and achieve biological modulation of physiological pathways. These findings imply that the unique binding patterns of these compounds may contribute to varying therapeutic efficacies and selectivities, highlighting their promising potential for modulating the functions of AKT and PI3K.

The literature partially confirms the results obtained from in silico studies. Particularly, luteolin, a bioactive flavone derivative present mainly in its shell, exerts breast cancer-inhibiting properties through an anti-angiogenesis mechanism by inhibiting VEGF production and its binding with the receptor^28^. In addition, it also downregulates epithelial-mesenchymal transition markers and lowers metastatic activity. Studies have shown that another compound, quercetin, reduces tumor weight by targeting VEGFR2 through the Akt/mTOR/P70S6K signaling pathway^30^. Emodin, another selected compound based on in silico experiments, inhibits cancer growth by suppressing the expression of MMP7, MMP9, VEGF, EMT, N-cadherin, b-catenin, and Snail based on the literature. It also inhibits the Wnt/b-catenin signaling pathway by downregulating target genes, including c-Myc, Cyclin-D1, and TCF4. In addition, endocrocin is reported to have anticancer properties, although there is a lack of available data about the possible mechanisms of its action^31^. Based on the in silico studies, we assumed that these 4 compounds could have important contributions to the overall promising anticancer properties of RO extract. Further in vitro and in vivo evaluation of their anticancer potential, both separate and with different combinations, are needed to confirm their role in the RO extract's anticancer properties and elucidate the role of combined treatment.

In conclusion, this study revealed the potential of the *R. obtusifolius* seed alcoholic extract as an adjunct therapy in cancer treatment, specifically in combination with the classical chemotherapeutic agent 5-fluorouracil. These findings pave the way for further investigations into the development of novel, targeted cancer treatment strategies that harness the potential of medicinal plants like *R. obtusifolius*.

### Limitations of the study

1. The current research primarily investigated herbal decoction. Future work will explore individual phytochemicals, especially those identified in silico studies, with the highest affinity for the PI3K/Akt pathway.
2. This study focused on the PI3K/Akt pathway. Future studies will expand the investigation to include other important signaling pathways, such as MAPK, Nrf2, and JAK/STAT.
3. The observed changes in the PI3K/Akt pathway have yet to be confirmed through in vivo studies. Future research will aim to validate these results using experimental models of different types of cancer in rats/mice.
4. The study did not elucidate the key members of the cell signaling pathways in different cell lines. Future studies will address this by examining these pathways across various cell lines to provide a more comprehensive understanding.

## Methods

### Chemicals and reagents

All chemicals were purchased from Sigma-Aldrich (USA) and Abcam (UK). Antibodies against TNFa (ab46087), VEGFa (ab193555), MMP-2 (ab92536), COX-2 (ab38898), PI3K and phosphorylated (p)-PI3K (ab191606), as well as ELISA kits for AKT and p-AKT (ab179463) were purchased from Abcam.

### Plant material

The *Rumex obtusifolius* L. seeds were harvested from the Tavush region of Armenia (1400–1600 m height above mean sea level) according to the protocol described before^32^. Dr. Narine Zakaryan identified plant material at the YSU Department of Botany and Mycology. Plant materials were deposited at the Herbarium of YSU, where the Voucher specimen serial number was given (ERCB 13208)**.** The collection of plant material complied with relevant institutional, national, and international guidelines and legislation. *Rumex obtusifolius* L., commonly known as broad-leaved dock, is an edible plant widely distributed and commonly found throughout Armenia. It is not on the list of Endangered species in Armenia (https://worldrainforests.com/biodiversity/en/armenia/EN.html /https://www.iucnredlist.org/search?query=Rumex%20obtusifolius%20&searchType=species / https://cites.org/eng/search?search_api_fulltext=Rumex+obtusifolius +). The plant is prevalent in natural settings and routinely collected by local populations for culinary purposes. There is no specific prohibition or regulatory constraint on the collection of this plant in Armenia, and it is a common sight at local markets, where it is sold after being gathered from the wild. This widespread availability and cultural integration into local diets supports the ethical sourcing and utilization of *Rumex obtusifolius* for research purposes under the conditions described in our study. For our research, we specifically collected only the seeds of *Rumex obtusifolius*. This collection method ensures minimal impact on the natural populations of the plant, as it does not involve uprooting or damaging the plants themselves. We ensure our research practices are sensitive to ecological and conservation concerns, even in cases where no formal collection restrictions exist. Our study strictly adheres to general ethical guidelines for botanical research despite the lack of specific regulations surrounding the collection of *Rumex obtusifolius* in Armenia.

### Plant crude extract

The grounded seeds were extracted by maceration with 96% ethanol at a 10:1 solvent-to-sample ratio (v/w). Stock solutions of 50 mg DW/mL crude ethanol extract were prepared as described earlier^33^. The percent yield was 10.60 ± 2.31%.

### Cell cultures

Human lung adenocarcinoma A549 cells were obtained from ATCC (cat # CCL-185) and maintained in DMEM medium supplemented with L-glutamine (2 mmol/L), sodium pyruvate (200 mg/L), fetal bovine serum (100 mL/L), and antibiotics (100 U/mL penicillin and 100 µg/L streptomycin). The cells were grown at 37 °C under a humidified atmosphere with 5% CO_2_ in a Biosmart (Biosan, Latvia) as described before^34^. Cultured cells were regularly examined for mycoplasma contamination using the Universal Mycoplasma Detection Kit from ATCC (Manassas, Virginia, USA).

### MTT cytotoxicity test

The growth-inhibiting properties of the *R. obtusifolius* ethanol extract and its combination with 5-FU were assessed in A549 cells using the MTT assay, as described previously^35^. For combination studies, the cells were seeded in 96-well plates and exposed to different concentrations of 5-FU in the absence or presence of 0.25 mg DW/mL of RO extract for 4, 24, or 72 h. Cell growth was assessed as described previously^36^. The results were calculated as the percentage of cell growth in the presence of the tested compounds, extracts, or their combinations, compared to control cells treated with the corresponding volume of solvent alone (1% EtOH in the final culture), whose growth was considered 100%. Three independent replicates of each treatment were performed with three technical replicates.

### ELISA of TNFa, VEGFa, COX-2, MMP-2, and Akt

A549 cells (2 × 10^5^) were cultured in 12-well plates and incubated for 24 h. After incubation, the cell medium (630 μL) was replaced, and the cells were treated with PBS and 1% ethanol solution (Control, A549C), 5-FU (40 μM), RO (0.25 mg/mL), and RO + 5- FU (0.25 mg/mL + 40 μM) for 24 h and then the culture medium was harvested. TNFa, VEGFa, and MMP-2 in the supernatant were quantified according to the manufacturer's instructions. Cells from each group were collected (trypsinized, neutralized, centrifuged), lysed on ice with lysis buffer, collected in a centrifuge tube, and further lysed for 10 min. The supernatant was collected after centrifugation at 13,000 × g for 10 min at 4 °C. Changes in the levels of COX-2 and Akt were measured using ELISA kits, according to the manufacturer's instructions. The protein concentration in the cell culture medium and lysates were measured using the Bradford method. Each test sample (70 μL) was added to three different passages in triplicate.

### Cells preparation for arginase, NOS, and NO activity, MDA analysis

A549 cells were seeded in 24-well (5 × 10^4^ cells per well) plates and incubated for 24 h. After incubation, the medium in the wells (450 μL) was refreshed. The cells were treated with 50 μL of the control or test compounds at the following final concentrations: PBS, 1% ethanol (Control, A549C), 5-FU (40 μM), RO (0.25 mg/mL), and RO + 5-FU (0.25 mg/mL + 40 μM). After 24 h, the supernatant was discarded. Cells from each group were collected (trypsinized, neutralized, centrifuged), lysed on ice with lysis buffer, collected in a centrifuge tube, and further lysed for 10 min. The supernatant was collected after centrifugation at 13,000 × g for 10 min at 4 °C. The levels of Nitrite anions, MDA, Arginase, and NOS were quantified according to the methods described below^14^. Each test sample (50 μL) was added to five passages in triplicate.

### NO quantity measurement

NO levels in the cell culture medium were determined as nitrite anions. The Griess assay was used as described before^37^. A total of 100 μL Griess reactant was added to 100 μL of each sample. The supernatants were transferred to the tubes containing pellets of cadmium and incubated at room temperature for 12 h to convert nitrate to nitrite. The samples’ absorbance was measured at λ = 550 nm, and the NO quantity was calculated based on a standard curve prepared with NaNO_2_.

### MDA assay

MDA quantity in the cell culture medium was determined by a colorimetric assay using the Ohkawa thiobarbituric acid-malondialdehyde method^38^.

### Arginase activity

The modified Diacetyl Monoxime colorimetric method assessed the arginase activity in A549 cell lysates^39^.

### NOS activity

Nitric oxide synthase activity (µmol citrulline/mg protein) in A549 cell lysates was measured by converting L-arginine to L-citrulline^40^. A total of 100 µl of the cell lysate was added to 200 mL of reaction mixture (50 mmol/L Tris buffer, pH 7.4, containing 10 mmol/L dithiothreitol (DTT), 10 µmol/L tetrahydrobiopterin (THB4), 10 µg/mL calmodulin, 1 mmol/L NADPH, 4 µmol/L flavin adenine dinucleotide (FAD), 4 µmol/L flavin mononucleotide (FMN), and 2 µmol/L L-arginine). The assay was carried out at 37 °C, and it was terminated with 2 mL of ice-cold stop buffer (20 mmol/L CH3COONa, pH 5.5, containing 2 mmol/L EDTA and 1 mmol/L L-citrulline). Assays were systematically performed with Ca^2+^ (1 mmol/L CaCl2) or without Ca^2+^ (0 mmol/L CaCl2) to measure total versus Ca^2+^-independent NOS activities. The Ca^2+^-dependent NOS activity was calculated as total NOS activity minus Ca^2+^-independent NOS activity. All assays were performed in triplicate on aliquoted samples (to avoid freezing/thawing cycles). The results were normalized for protein content.

### Phospho-PI 3 kinase p85 + total in-cell ELISA assay

A549 cells (1.5 × 10^4^ cells per well) were seeded in the 96-well plates treated for tissue culture. After 24 h incubation, the cell medium (180 μL) was refreshed. The cells were treated with 20μL of the control or test compounds at the following final concentrations: PBS, 1% ethanol solution (Control, A549C), 5-FU (40 μM), RO (0.25 mg/mL), and RO + 5-FU (0.25 mg/mL + 40 μM). The calculations during the seeding of the cells were done in a way that reached approximately 80% confluency at fixation time. After 24 h, the medium was discarded, and the cells were fixed with 100 µL of 4% formaldehyde in PBS. Crystal Violet was used to stain cells for normalizing readings in 450 nm for Phospho-PI 3 kinase p85 + Total. The OD450 readings were corrected for cell number by dividing the OD450 reading for a given well by the OD595 reading. This relative cell number was then used to normalize each reading. Total and phospho-PI 3 kinase p85 were each assayed in triplicate using the phospho- and total PI 3 Kinase p85 antibodies included in the PI 3 Kinase Kit. According to the manufacturer's instructions, phospho-PI 3 kinase p85 and Total PI3K levels were measured using an In-Cell ELISA kit (ab207484).

### Caspase-3/CPP32 colorimetric assay

A549 cells (5 × 10^5^ cells per well) were cultured in 6-well plates and incubated for 24 h. Then, the cell medium (900 μL) was refreshed, and the cells were treated with 10 μL of PBS + 1% ethanol solution (control, A549C) or test compounds at the following final concentrations: 5-FU (40 μM), RO (0.25 mg/mL), and RO + 5- FU (0.25 mg/mL + 40 μM). After 24 h, the cells were harvested. Each test sample (100 μL) was added to three different passages in triplicate. The cells were resuspended in 50 µL of chilled Cell Lysis Buffer and incubated on ice for 10 min. Then, the cell lysate was centrifuged for 1 min (10,000 × g). Next, the supernatant (cytosolic extract) was transferred to a fresh tube and put on ice for immediate assay. Fold-increase in CPP32 activity has been determined by comparing these results with the level of the uninduced control. Optical density values were corrected, taking into account the number of cells. All the steps were performed according to the protocol presented in the Caspase-3/CPP32 Colorimetric Assay Kit (K106, BioVision) instructions.

### Analysis of apoptosis by Hoechst 33258 staining

The percentage of apoptotic cells was evaluated as previously described^41^. A549 cells (2 × 10^5^ cells/mL) were treated with vehicle or 5-FU (40 μM), RO (0.25 mg/mL), and RO + 5- FU (0.25 mg/mL + 40 μM) for 24 h, respectively. After the treatment, the cells were washed with PBS and fixed with 4% paraformaldehyde in PBS for 10 min. Then, the cells were washed twice with PBS for 5 min and stained with Hoechst 33258 reagent (10 μg/mL) for 10 min at room temperature in the dark. Then, the cells were washed with PBS and analyzed under a fluorescence microscope (× 250 magnification) (Zeiss, Germany). The Hoechst 33258 staining identifies apoptotic cells based on nuclear morphology. Cells with typical morphological changes, such as karyopyknosis, hyperfluorescence, nuclear fragmentation, and apoptotic bodies, were considered apoptotic. All variants were examined in duplicate. For each treatment variant, 500 cells were scored, and the percentage of apoptotic cells was calculated as follows: % apoptotic cells = (the number of apoptotic cells/500 cells)*100.

### Preparation of protein structures

The crystallographic structures of PI3K and AKT were procured from the Protein Data Bank (PDB) database (https://www.rcsb.org/), using the identifiers 6AUD and 2JDO, respectively. Visualization and preliminary assessment of these structures were performed with the PyMOL Molecular Graphics System (Schrödinger, LLC). The retrieved crystallographic structures were subject to preprocessing, which involved removing extraneous entities, such as water molecules, ions, and other non-protein moieties contained within the structures. Simultaneously, the ligands co-crystallized with each protein structure were separated and retained for redocking validation experiments. The resulting streamlined protein structures were then used for docking explorations. The extracted ligands, on the other hand, were reserved for ensuing redocking studies as controls.

### Docking

Ligand docking and binding site analysis with PyMOL and Autodock/Vina were used for docking^42^. The protein and ligand structures were prepared using Autodock Tools^43^. During a typical procedure, the "exhaustiveness" parameter was calibrated to 8, and standard parameters suggested by the program creators were used to ensure the fidelity of the results. The compounds were sorted based on their binding strengths. The 2D binding mode analysis of best docking scores was performed using LigPlot + software (EMBL-EBI).

### Statistic analysis

All the results are presented as the means ± SEM. We analyzed the data either by one-way ANOVA or by its non-parametric analog Kruskal–Wallis test based on the normality test performed followed by Dunn's test, which was used to evaluate the statistical significance of the TNFa, VEGFa, MMP-2, COX-2, arginase, NOS, MDA, nitrite anions, Caspase-3, and apoptosis rate results. The significance of the results obtained for PI3K and Akt was assessed using two-way ANOVA and Tukey's multiple comparisons tests. The statistical analyses were performed using GraphPad Prism 8 software (San Diego, CA, USA), and a significance level of *p* < 0.05 was deemed statistically significant.

### Supplementary Information

Supplementary Information.

## Data Availability

The data used to support the findings of this study are included in the articles.

## Abbreviations

Akt: Protein kinase B
ANOVA: Analysis of variance
Bcl-2: B-cell lymphoma-2
COX-2: Cyclooxygenase-2
CoQ0: Coenzyme Q0
ELISA: Enzyme-linked immunosorbent assay
EMT: Epithelial–mesenchymal transition
FAD: Flavin adenine dinucleotide
FMN: Flavin mononucleotide
HBA: Hydrogen bond acceptors
HBD: Hydrogen bond donors
IκBα: An inhibitor of NF-κBα
IL-6: Interleukin-6
MAPK: Mitogen-activated protein kinase
MTT: 3-(4,5-Dimethylthiazol-2-yl)-2,5-diphenyl tetrazolium bromide
MMP-2: Matrix metalloproteinase-2
MW: Molecular weight
NOXs: NADPH oxidases
NF-κB: Nuclear factor-κB
NOS: Nitric oxide synthase
NSCLC: Non-small cell lung cancer
Nrf2: The transcription factor nuclear factor erythroid 2-related factor 2
PI3K: Phosphoinositol-3-kinase
PG E2: Prostaglandin E2
RO: *Rumex obtusifolius* Extract
ROS: Reactive oxygen species
TNF-α: Tumor necrosis factor alpha
THB4: Tetrahydrobiopterin
VEGF: Vascular endothelial growth factor
