# Supplementary material for: 5-fluorouracil and Rumex obtusifolius extract combination trigger A549 cancer cell apoptosis: uncovering PI3K/Akt inhibition by in vitro and in silico approaches
Source: Sci Rep. 2024 Jun 25;14:14676. doi: 10.1038/s41598-024-65816-5 (PMC11199614; doi:10.1038/s41598-024-65816-5)
Supplement: Supplementary file 1 — Supplementary Information. [file 41598_2024_65816_MOESM1_ESM.docx]

**Supplementary table 1. Phytochemicals tentatively identified by LC-Q-Orbitrap-HRMS in *R. obtusifolius* seed extract**

| **no** | **Compound Group** | **Tentative identification** | **RT [min]** | **Molecular formula** | **Molecular Weight** | **Theoretical (m/z)** | **Observed (m/z)** | **Mass Error (ppm)** | **Fragments (m/z)** |
| --- | --- | --- | --- | --- | --- | --- | --- | --- | --- |
| 1 | HT* | Glucogallic acid isomer | 2.13 | C_13_H_16_O_10_ | 332.07435 | 331.066525 | 331.067015 | -1.48 | 125.02; 151.00; 169.01; 211.02 |
| 2 | HT | Glucogallic acid isomer | 2.76 | C_13_H_16_O_10_ | 332.07435 | 331.066525 | 331.067015 | -1.48 | 125.02; 151.00; 169.01; 211.02 |
| 3 | CT | Procyanidin trimer | 2.95 | C_45_H_38_O_18_ | 866.20582 | 865.197995 | 865.198735 | -0.85 | 287.06; 413.09; 575.12; 695.14 |
| 4 | HBA | Protocatechuic acid glucoside | 3.05 | C_13_H_16_O_9_ | 316.079435 | 315.07161 | 315.072205 | -1.89 | 109.00; 153.02 |
| 5 | HBA | Hydroxybenzoic acid glucoside | 3.22 | C_13_H_16_O_8_ | 300.08452 | 299.076695 | 299.077045 | -1.17 | 93.03; 137.02 |
| 6 | CT | Procyanidin trimer | 3.33 | C_45_H_38_O_18_ | 866.20582 | 865.197995 | 865.198735 | -0.85 | 287.06; 425.09; 575.12; 695.14 |
| 7 | HBA | Gallic acid | 3.39 | C_7_H_6_O_5_ | 170.021525 | 169.0137 | 169.013805 | -0.62 | 69.03; 79.02; 97.03; 125.02 |
| 8 | HT | Glucogallic acid isomer | 3.69 | C_13_H_16_O_10_ | 332.07435 | 331.066525 | 331.067015 | -1.48 | 125.02; 169.01 |
| 9 | HBA | Protocatechuic acid glucoside isomer | 4.00 | C_13_H_16_O_9_ | 316.079435 | 315.07161 | 315.072205 | -1.89 | 108.02; 152.01; 153.02 |
| 10 | HBA | Protocatechuic acid glucoside isomer | 4.26 | C_13_H_16_O_9_ | 316.079435 | 315.07161 | 315.072205 | -1.89 | 109.00; 153.02 |
| 11 | F-3-ol | Epi/catechin hexoside | 4.43 | C_21_H_24_O_11_ | 452.131865 | 451.12404 | 451.124515 | -1.05 | 245.08; 289.07 |
| 12 | HBA | Methoxysalicylic acid | 4.68 | C_8_H_8_O_4_ | 168.04226 | 167.034435 | 167.034655 | -1.31 | 93.03; 109.03; 122.04; 137.02 |
| 13 | HBA | Hydroxybenzoic acid glucoside isomer | 4.77 | C_13_H_16_O_8_ | 300.08452 | 299.076695 | 299.076965 | -0.90 | 137.02 |
| 14 | HBA | Hydroxybenzoic acid glucoside isomer | 5.08 | C_13_H_16_O_8_ | 300.08452 | 299.076695 | 299.076965 | -0.90 | 137.02 |
| 15 | HCA | 3-Caffeoylquinic acid | 5.26 | C_16_H_18_O_9_ | 354.095085 | 353.08726 | 353.087675 | -1.17 | 135.04; 179.03; 191.06 |
| 16 | HCA | Caffeoyl hexoside | 5.26 | C_15_H_18_O_9_ | 342.095085 | 341.08726 | 341.087555 | -0.86 | 135.04; 179.03 |
| 17 | HCA | p-Coumaroyl hexoside | 5.43 | C_15_H_18_O_8_ | 326.10017 | 325.092345 | 325.092755 | -1.26 | 119.05; 163.04 |
| 18 | HBA | 3,4,5-Trimethoxybenzoic acid | 5.56 | C_10_H_12_O_5_ | 212.068475 | 211.06065 | 211.060365 | 1.35 | 109.03; 148.02; 153.05 |
| 19 | F-3-ol | Epi/catechin hexoside | 5.62 | C_21_H_24_O_11_ | 452.131865 | 451.12404 | 451.124515 | -1.05 | 245.08; 289.07 |
| 20 | HBA | Protocatechuic acid | 5.75 | C_7_H_6_O_4_ | 154.02661 | 153.018785 | 153.019005 | -1.44 | 108.02; 109.03 |
| 21 | CT | Procyanidin dimer | 6.07 | C_30_H_26_O_12_ | 578.14243 | 577.134605 | 577.135235 | -1.09 | 125.02; 289.07; 407.08 |
| 22 | HCA | Feruloyl hexoside | 6.37 | C_16_H_20_O_9_ | 356.110735 | 355.10291 | 355.103265 | -0.99 | 134.03; 193.05 |
| 23 | HT | Hamamelitannin | 6.42 | C_20_H_20_O_14_ | 484.08531 | 483.077485 | 483.077955 | -0.97 | 169.01; 271.05; 313.06; 439.09 |
| 24 | FO | Quercetin-diglucoside | 6.43 | C_27_H_30_O_17_ | 626.148305 | 625.14048 | 625.140995 | -0.83 | 301.04; 463.09 |
| 25 | CT | Procyanidin tetramer one A-type linkage | 6.72 | C_60_H_48_O_24_ | 1152.25356 | 1151.245735 | 1151.246855 | -0.97 | 573.10; 711.14; 863.18 |
| 26 | HCA | Caffeoyl hexoside | 6.99 | C_15_H_18_O_9_ | 342.095085 | 341.08726 | 341.087315 | -0.16 | 135.04; 179.03 |
| 27 | HCA | p-Coumaroylquinic acid | 7.00 | C_16_H_18_O_8_ | 338.10017 | 337.092345 | 337.092625 | -0.83 | 119.05; 163.04; 191.06 |
| 28 | CT | Procyanidin dimer | 7.05 | C_30_H_26_O_12_ | 578.14243 | 577.134605 | 577.135225 | -1.07 | 125.02; 289.07; 407.08 |
| 29 | HCA | p-Coumaroyl hexoside | 7.29 | C_15_H_18_O_8_ | 326.10017 | 325.092345 | 325.092865 | -1.59 | 119.05; 163.04 |
| 30 | HCA | 5-Caffeoylquinic acid | 7.36 | C_16_H_18_O_9_ | 354.095085 | 353.08726 | 353.087675 | -1.17 | 191.06 |
| 31 | CT | Procyanidin trimer | 7.39 | C_45_H_38_O_18_ | 866.20582 | 865.197995 | 865.198915 | -1.06 | 287,06; 425,09; 577,14; 695,14 |
| 32 | HT | Hydroxy-methoxyphenyl-O-(O-galloyl)-hexose | 7.47 | C_20_H_22_O_12_ | 454.11113 | 453.103305 | 453.103825 | -1.15 | 169.01; 313.06 |
| 33 | CT | Procyanidin trimer | 7.53 | C_45_H_38_O_18_ | 866.20582 | 865.197995 | 865.198915 | -1.06 | 287.06; 425.09; 577.14; 695.14 |
| 34 | CT | Procyanidin tetramer monogallate | 7.58 | C_67_H_54_O_28_ | 1306.28017 | 1305.272345 | 1305.273455 | -0.85 | 652.13 (M − 2H)^2−^ |
| 35 | F-3-ol | Catechin | 7.64 | C_15_H_14_O_6_ | 290.07904 | 289.071215 | 289.071515 | -1.04 | 245.08; 205.05 |
| 36 | FNO | Taxifolin | 7.65 | C_15_H_12_O_7_ | 304.058305 | 303.05048 | 303.050445 | 0.12 | 109.03; 125.02; 137.02 |
| 37 | FNN | Eriodictyol | 7.66 | C_15_H_12_O_6_ | 288.06339 | 287.055565 | 287.055905 | -1.18 | 109.03; 123.04; 137.02; 151.04 |
| 38 | F-3-ol | Gallocatechin | 7.77 | C_15_H_14_O_7_ | 306.073955 | 305.06613 | 305.066555 | -1.39 | 125.02; 137.02; 161.02 |
| 39 | HCA | Feruloylquinic acid | 7.78 | C_17_H_20_O_9_ | 368.110735 | 367.10291 | 367.103275 | -0.99 | 134.03; 193.05 |
| 40 | HBA | Hydroxybenzoic acid | 7.95 | C_7_H_6_O_3_ | 138.031695 | 137.02387 | 137.024055 | -1.35 | - |
| 41 | CT | Procyanidin pentamer one A-type linkage | 8.23 | C_75_H_60_O_30_ | 1440.31695 | 1439.309125 | 1439.311905 | -1.93 | 719,15 (M − 2H)2− |
| 42 | HCA | Feruloyl glucose | 8.24 | C_16_H_20_O_9_ | 356.110735 | 355.10291 | 355.103275 | -1.03 | 134,03; 178,03; 193,05 |
| 43 | CT | Procyanidin heptamer | 8.25 | C_105_H_86_O_42_ | 2018.45938 | 2017.451555 | 2017.452285 | -0.36 | 1008,22 (M − 2H)2− |
| 44 | CT | Procyanidin trimer monogallate | 8.33 | C_52_H_42_O_22_ | 1018.21678 | 1017.208955 | 1017.209845 | -0.87 | 287,06; 575,12; 729,15; 847,16 |
| 45 | HCA | p-Coumaroyl hexoside | 8.35 | C_15_H_18_O_8_ | 326.10017 | 325.092345 | 325.092685 | -1.04 | 119,05; 145,05; 163,04 |
| 46 | CT | Procyanidin pentamer one A-type linkage monogallate | 8.41 | C_82_H_64_O_34_ | 1592.32791 | 1591.320085 | 1591.321915 | -1.15 | 795.66 (M − 2H)^2−^ |
| 47 | CT | Procyanidin trimer | 8.43 | C_45_H_38_O_18_ | 866.20582 | 865.197995 | 865.198915 | -1.06 | 287.06; 425.09; 577.14; 695.14 |
| 48 | CT | Procyanidin dimer | 8.51 | C_30_H_26_O_12_ | 578.14243 | 577.134605 | 577.135255 | -1.13 | 125.02; 289,07; 407.08 |
| 49 | CT | Procyanidin dimer one A-type linkage | 8.61 | C_30_H_24_O_12_ | 576.12678 | 575.118955 | 575.119855 | -1.56 | 289.07; 407.08 |
| 50 | CT | Procyanidin pentamer | 8.78 | C_75_H_62_O_30_ | 1442.3326 | 1441.324775 | 1441.326915 | -1.48 | 720.16 (M − 2H)^2−^ |
| 51 | CT | Procyanidin pentamer one A-type linkage | 8.93 | C_75_H_60_O_30_ | 1440.31695 | 1439.309125 | 1439.311655 | -1.76 | 719.15 (M − 2H)^2−^ |
| 52 | CT | Procyanidin haxamer one A-type linkage monogallate | 8.94 | C_97_H_76_O_40_ | 1880.3913 | 1879.383475 | 1879.385635 | -1.15 | 939.19 (M − 2H)^2−^ |
| 53 | HCA | p-Coumaroyl glucose | 9.11 | C_15_H_18_O_8_ | 326.10017 | 325.092345 | 325.092715 | -1.14 | 145.03; 163.04 |
| 54 | HCA | Sinapoylquinic acid | 9.18 | C_18_H_22_O_10_ | 398.1213 | 397.113475 | 397.113865 | -0.98 | 189.06 |
| 55 | CT | Procyanidin pentamer digallate | 9.21 | C_89_H_70_O_38_ | 1746.35452 | 1745.346695 | 1745.347065 | -0.21 | 872.17 (M − 2H)^2−^ |
| 56 |  | Unknown | 9.22 | C_15_H_12_O_6_ | 288.06339 | 287.055565 | 287.055905 | -1.18 | 123.04; 151.04; 203.07 |
| 57 | HBA | Ethyl vanillin glucoside | 9.25 | C_15_H_20_O_8_ | 328.11582 | 327.107995 | 327.108385 | -1.19 | 165.05 |
| 58 | CT | Procyanidin tetramer one A-type linkage monogallate | 9.31 | C_67_H_52_O_28_ | 1304.26452 | 1303.256695 | 1303.258555 | -1.43 | 651.13 (M − 2H)^2−^ |
| 59 | HCA | p-Coumaroyl glycolic acid | 9.37 | C_11_H_10_O_5_ | 222.052825 | 221.045 | 221.044645 | 1.61 | 109.03; 119.05; 149.06; 177.05 |
| 60 | F-3-ol | Epicatechin | 9.40 | C_15_H_14_O_6_ | 290.07904 | 289.071215 | 289.071535 | -1.11 | 205.05; 245.08 |
| 61 | CT | Procyanidin trimer one A-type linkage | 9.46 | C_45_H_36_O_18_ | 864.19017 | 863.182345 | 863.183415 | -1.24 | 289.07; 411.07; 573.10; 711.14 |
| 62 | CT | Procyanidin pentamer monogallate | 9.57 | C_82_H_66_O_34_ | 1594.34356 | 1593.335735 | 1593.338025 | -1.44 | 796.17 (M − 2H)^2−^ |
| 63 | CT | Procyanidin heptamer one A-type linkage | 9.64 | C_105_H_84_O_42_ | 2016.44373 | 2015.435905 | 2015.437275 | -0.68 | 1007.21 (M − 2H)^2−^ |
| 64 | AQ | Glucoaloe emodin | 9.76 | C_21_H_22_O_11_ | 450.116215 | 449.10839 | 449.108945 | -1.24 | 259.06; 269.05; 287.06 |
| 65 | CT | Procyanidin trimer monogallate | 9.79 | C_52_H_42_O_22_ | 1018.21678 | 1017.208955 | 1017.210215 | -1.24 | 287.06; 575.12; 729.15; 847.16 |
| 66 | CT | Procyanidin tetramer monogallate | 9.87 | C_67_H_54_O_28_ | 1306.28017 | 1305.272345 | 1305.274935 | -1.98 | 652.13 (M − 2H)^2−^ |
| 67 | HCA | p-Coumaroylquinic acid | 9.87 | C_16_H_18_O_8_ | 338.10017 | 337.092345 | 337.092715 | -1.09 | 191.06; 163.04 |
| 68 | CT | Procyanidin tetramer one A-type linkage | 9.96 | C_60_H_48_O_24_ | 1152.25356 | 1151.245735 | 1151.246855 | -0.97 | 573.10; 699.14; 863.18 |
| 69 | CT | Procyanidin heptamer one A-type linkage | 9.99 | C_105_H_84_O_42_ | 2016.44373 | 2015.435905 | 2015.437395 | -0.74 | 1007.21 (M − 2H)^2−^ |
| 70 | CT | Procyanidin trimer one A-type linkage | 10.08 | C_45_H_36_O_18_ | 864.19017 | 863.182345 | 863.183415 | -1.24 | 289.07; 411.07; 573.10; 711.14 |
| 71 | HT | Galloylsalidroside | 10.20 | C_21_H_24_O_11_ | 452.131865 | 451.12404 | 451.124585 | -1.21 | 169.01; 313.06; 271.05 |
| 72 | CT | Procyanidin pentamer | 10.33 | C_75_H_62_O_30_ | 1442.3326 | 1441.324775 | 1441.326795 | -1.40 | 720.16 (M − 2H)^2−^ |
| 73 | CT | Procyanidin haxamer one A-type linkage monogallate | 10.45 | C_97_H_76_O_40_ | 1880.3913 | 1879.383475 | 1879.385515 | -1.08 | 939.19 (M − 2H)^2−^ |
| 74 | FO | Isorhamnetin hexoside | 10.52 | C_22_H_22_O_12_ | 478.11113 | 477.103305 | 477.103795 | -1.03 | 314.04 |
| 75 | CT | Procyanidin tetramer one A-type linkage | 10.56 | C_60_H_48_O_24_ | 1152.25356 | 1151.245735 | 1151.246965 | -1.07 | 573.10; 863.18; 999.20 |
| 76 | CT | Procyanidin tetramer | 10.70 | C_60_H_50_O_24_ | 1154.26921 | 1153.261385 | 1153.262085 | -0.61 | 287.06; 575.12; 863.18 |
| 77 | CT | Procyanidin dimer | 10.74 | C_30_H_26_O_12_ | 578.14243 | 577.134605 | 577.134945 | -0.59 | 125.02; 289.07; 407.08 |
| 78 | CT | Procyanidin dimer monogallate | 10.77 | C_37_H_30_O_16_ | 730.15339 | 729.145565 | 729.146485 | -1.26 | 289.07; 407.08 |
| 79 | CT | Procyanidin dimer monogallate | 10.86 | C_37_H_30_O_16_ | 730.15339 | 729.145565 | 729.1465 | -1.28 | 289.70; 407.08; 577.14 |
| 80 |  | Unknown | 10.97 | C_14_H_14_O_6_ | 278.07904 | 277.071215 | 277.071415 | -0.72 | 189.06; 233.03 |
| 81 | CT | Procyanidin trimer | 10.99 | C_45_H_38_O_18_ | 866.20582 | 865.197995 | 865.198605 | -0.70 | 289.07; 425.09; 577.14; 695.14 |
| 82 | CT | Procyanidin pentamer one A-type linkage monogallate | 11.01 | C_82_H_64_O_34_ | 1592.32791 | 1591.320085 | 1591.322285 | -1.38 | 795.66 (M − 2H)^2−^ |
| 83 | CT | Procyanidin trimer monogallate | 11.05 | C_52_H_42_O_22_ | 1018.21678 | 1017.208955 | 1017.209795 | -0.83 | 287.06; 577.14; 727.13; 865.19 |
| 84 | CT | Procyanidin pentamer | 11.07 | C_75_H_62_O_30_ | 1442.3326 | 1441.324775 | 1441.32302 | 1.22 | 720.16 (M − 2H)^2−^ |
| 85 | HCA | p-Coumaroylquinic acid | 11.22 | C_16_H_18_O_8_ | 338.10017 | 337.092345 | 337.092895 | -1.63 | 191.06 |
| 86 | CT | Procyanidin pentamer digallate | 11.27 | C_89_H_70_O_38_ | 1746.35452 | 1745.346695 | 1745.348645 | -1.12 | 872.17 (M − 2H)^2−^ |
| 87 | CT | Procyanidin tetramer one A-type linkage monogallate | 11.28 | C_67_H_52_O_28_ | 1304.26452 | 1303.256695 | 1303.258555 | -1.43 | 651.13 (M − 2H)^2−^ |
| 88 |  | Unknown | 11.38 | C_16_H_28_O_6_ | 316.18859 | 315.180765 | 315.181065 | -0.95 | 177.02; 191.03; 235.06 |
| 89 | CT | Procyanidin pentamer monogallate | 11.42 | C_82_H_66_O_34_ | 1594.34356 | 1593.335735 | 1593.338275 | -1.59 | 796.17 (M − 2H)^2−^ |
| 90 | CT | Procyanidin pentamer | 11.44 | C_75_H_62_O_30_ | 1442.3326 | 1441.324775 | 1441.326795 | -1.40 | 720.16 (M − 2H)^2−^ |
| 91 | LN | Lariciresinol hexoside | 11.58 | C_26_H_34_O_11_ | 522.210115 | 521.20229 | 521.202955 | -1.28 | 359.15 |
| 92 | CT | Procyanidin tetramer | 11.59 | C_60_H_50_O_24_ | 1154.26921 | 1153.261385 | 1153.262155 | -0.67 | 287.06; 575.12; 865.20; 1027.23 |
| 93 | CT | Procyanidin haxamer monogallate | 11.66 | C_97_H_78_O_40_ | 1882.40695 | 1881.399125 | 1881.401865 | -1.46 | 940.20 (M − 2H)^2−^ |
| 94 | LN | Cyclo-olivil | 11.70 | C_20_H_24_O_7_ | 376.152205 | 375.14438 | 375.144905 | -1.40 | 345.10; 360.12 |
| 95 | FNN | Eriodictyol-glucoside | 11.77 | C_21_H_22_O_11_ | 450.116215 | 449.10839 | 449.109025 | -1.41 | 286.05 |
| 96 | CT | Procyanidin trimer monogallate | 11.78 | C_52_H_42_O_22_ | 1018.21678 | 1017.208955 | 1017.210215 | -1.24 | 287.06; 577.14; 727.13; 865.19 |
| 97 | CT | Procyanidin tetramer trigallate | 11.93 | C_81_H_62_O_36_ | 1610.30209 | 1609.294265 | 1609.295545 | -0.79 | 804.14 (M − 2H)^2−^ |
| 98 | CT | Procyanidin tetramer one A-type linkage monogallate | 11.94 | C_67_H_52_O_28_ | 1304.26452 | 1303.256695 | 1303.258555 | -1.43 | 651.13 (M − 2H)^2−^ |
| 99 | CT | Procyanidin tetramer digallate | 11.95 | C_74_H_58_O_32_ | 1458.29113 | 1457.283305 | 1457.285535 | -1.53 | 728,14 (M − 2H)2− |
| 100 | CT | Procyanidin haxamer monogallate | 12.02 | C_97_H_78_O_40_ | 1882.40695 | 1881.399125 | 1881.401865 | -1.46 | 940.20 (M − 2H)^2−^ |
| 101 | LN | Nortrachelogenin | 12.02 | C_20_H_22_O_7_ | 374.136555 | 373.12873 | 373.129305 | -1.54 | 297.08; 328.10; 343.12 |
| 102 | FNN | Eriodictyol-glucoside | 12.06 | C_21_H_22_O_11_ | 450.116215 | 449.10839 | 449.109005 | -1.37 | 286.05 |
| 103 | CT | Procyanidin haxamer | 12.07 | C_90_H_74_O_36_ | 1730.39599 | 1729.388165 | 1729.390155 | -1.15 | 864.19 (M − 2H)^2−^ |
| 104 | CT | Procyanidin pentamer one A-type linkage monogallate | 12.12 | C_82_H_64_O_34_ | 1592.32791 | 1591.320085 | 1591.323135 | -1.92 | 795.66 (M − 2H)^2−^ |
| 105 | LN | Lariciresinol hexoside | 12.18 | C_26_H_34_O_11_ | 522.210115 | 521.20229 | 521.202465 | -0.34 | 359.15 |
| 106 | FN | Chrysoeriol hexoside | 12.20 | C_22_H_22_O_11_ | 462.116215 | 461.10839 | 461.109125 | -1.59 | 254.06; 298.05 |
| 107 | FO | Quercetin-O-galloyl-hexoside | 12.25 | C_28_H_24_O_16_ | 616.10644 | 615.098615 | 615.099425 | -1.32 | 301.04; 463.09 |
| 108 | CT | Procyanidin trimer digallate | 12.36 | C_59_H_46_O_26_ | 1170.22774 | 1169.219915 | 1169.221815 | -1.62 | 881.16; 999.17; 1043.19; 1151.21 |
| 109 | FNN | Naringenin hexoside | 12.43 | C_21_H_22_O_10_ | 434.1213 | 433.113475 | 433.113735 | -0.60 | 151,00; 271,06 |
| 110 | CT | Procyanidin hexamer trigallate | 12.44 | C_111_H_86_O_48_ | 2186.42887 | 2185.421045 | 2185.421765 | -0.33 | 1092.21 (M − 2H)^2−^ |
| 111 | LN | Longifloroside B | 12.49 | C_27_H_36_O_13_ | 568.215595 | 567.20777 | 567.208555 | -1.38 | 329.14; 341.15; 359.15; 521.20 |
| 112 | FO | Quercetin rutinoside | 12.51 | C_27_H_30_O_16_ | 610.15339 | 609.145565 | 609.146245 | -1.12 | 301.04 |
| 113 | CT | Procyanidin tetramer trigallate | 12.53 | C_81_H_62_O_36_ | 1610.30209 | 1609.294265 | 1609.295545 | -0.79 | 804.14 (M − 2H)^2−^ |
| 114 | FO | Quercetin-O-galloyl-hexoside | 12.54 | C_28_H_24_O_16_ | 616.10644 | 615.098615 | 615.099245 | -1.02 | 301.04; 463.09 |
| 115 | FN | Luteolin-rutinoside | 12.57 | C_27_H_30_O_15_ | 594.158475 | 593.15065 | 593.151425 | -1.31 | 285.4 |
| 116 | CT | Procyanidin tetramer trigallate | 12.62 | C_81_H_62_O_36_ | 1610.30209 | 1609.294265 | 1609.296405 | -1.33 | 804.14 (M − 2H)^2−^ |
| 117 | CT | Procyanidin pentamer trigallate | 12.63 | C_96_H_74_O_42_ | 1898.36548 | 1897.357655 | 1897.359875 | -1.17 | 948.18 (M − 2H)^2−^ |
| 118 | CT | Procyanidin trimer monogallate | 12.64 | C_52_H_42_O_22_ | 1018.21678 | 1017.208955 | 1017.209415 | -0.45 | 287.06; 575.12; 729.15; 891.18 |
| 119 | CT | Procyanidin pentamer one A-type linkage | 12.65 | C_75_H_60_O_30_ | 1440.31695 | 1439.309125 | 1439.311535 | -1.67 | 719.15 (M − 2H)^2−^ |
| 120 | CM | Methoxycoumarin-acetic acid | 12.66 | C_12_H_10_O_5_ | 234.052825 | 233.045 | 233.044925 | 0.32 | 147.04; 189.06 |
| 121 | CT | Procyanidin pentamer one A-type linkage monogallate | 12.77 | C_82_H_64_O_34_ | 1592.32791 | 1591.320085 | 1591.322895 | -1.76 | 795.66 (M − 2H)^2−^ |
| 122 | CT | Procyanidin tetramer one A-type linkage monogallate | 12.78 | C_67_H_52_O_28_ | 1304.26452 | 1303.256695 | 1303.258555 | -1.43 | 651.13 (M − 2H)^2−^ |
| 123 |  | Unknown | 12.84 | C_28_H_30_N_4_O_11_ | 598.191111 | 597.183286 | 597.182925 | 0.60 | 357.10; 387.11; 417.12 |
| 124 | CT | Procyanidin dimer digallate | 12.92 | C_44_H_34_O_20_ | 882.16435 | 881.156525 | 881.157565 | -1.18 | 287.06; 407.08; 559.13; 729.15 |
| 125 | HBA | Benzoylmalic acid | 12.97 | C_11_H_10_O_6_ | 238.04774 | 237.039915 | 237.039825 | 0.38 | 107.05; 149.06 |
| 126 | FNN | Naringenin hexoside | 12.99 | C_21_H_22_O_10_ | 434.1213 | 433.113475 | 433.113865 | -0.90 | 271.06 |
| 127 | CT | Procyanidin tetramer trigallate | 13.02 | C_81_H_62_O_36_ | 1610.30209 | 1609.294265 | 1609.296885 | -1.63 | 804.14 (M − 2H)^2−^ |
| 128 | CT | Procyanidin dimer | 13.04 | C_30_H_26_O_12_ | 578.14243 | 577.134605 | 577.135195 | -1.02 | 125.02; 289.07; 407.08 |
| 129 | CT | Procyanidin trimer one A-type linkage monogallate | 13.07 | C_52_H_40_O_22_ | 1016.20113 | 1015.193305 | 1015.194355 | -1.03 | 289.07; 451.11; 573.10; 711.14; 863.18 |
| 130 | CT | Procyanidin tetramer one A-type linkage | 13.24 | C_60_H_48_O_24_ | 1152.25356 | 1151.245735 | 1151.246965 | -1.07 | 573.10; 863.18; 999.20 |
| 131 | HBA | Ellagic acid | 13.32 | C_14_H_6_O_8_ | 302.00627 | 300.998445 | 300.998685 | -0.79 | 229.01; 257.01; 284.00 |
| 132 | CT | Procyanidin tetramer digallate | 13.36 | C_74_H_58_O_32_ | 1458.29113 | 1457.283305 | 1457.285535 | -1.53 | 728.14 (M − 2H)^2−^ |
| 133 | FO | Quercetin-hexoside | 13.39 | C_21_H_20_O_12_ | 464.09548 | 463.087655 | 463.088115 | -0.99 | 301.04 |
| 134 | FN | Chrysoeriol hexoside | 13.40 | C_22_H_22_O_11_ | 462.116215 | 461.10839 | 461.109155 | -1.66 | 254.06; 298.05; 299.04 |
| 135 | CT | Procyanidin tetramer digallate | 13.47 | C_74_H_58_O_32_ | 1458.29113 | 1457.283305 | 1457.285535 | -1.53 | 728,14 (M − 2H)2− |
| 136 | CT | Procyanidin trimer monogallate | 13.51 | C_52_H_42_O_22_ | 1018.21678 | 1017.208955 | 1017.209605 | -0.64 | 287.06; 575.12; 729.15; 865.15 |
| 137 | FO | Quercetin-glucuronide | 13.52 | C_21_H_18_O_13_ | 478.074745 | 477.06692 | 477.067045 | -0.26 | 301.04 |
| 138 | F-3-ol | (epi)Catechin monogallate | 13.62 | C_22_H_18_O_10_ | 442.09 | 441.082175 | 441.082605 | -0.97 | 125.02; 169.01; 245.08; 289.07 |
| 139 | CT | Procyanidin trimer trigallate | 13.70 | C_66_H_50_O_30_ | 1322.2387 | 1321.230875 | 1321.232805 | -1.46 | 660.11 (M − 2H)^2−^ |
| 140 | CT | Procyanidin trimer one A-type linkage monogallate | 13.74 | C_52_H_40_O_22_ | 1016.20113 | 1015.193305 | 1015.194285 | -0.97 | 287.06; 451.10; 575.12; 729.15; 847.16 |
| 141 | CT | Procyanidin pentamer digallate | 13.82 | C_89_H_70_O_38_ | 1746.35452 | 1745.346695 | 1745.348645 | -1.12 | 872.17 (M − 2H)^2−^ |
| 142 | CT | Procyanidin pentamer trigallate | 13.90 | C_96_H_74_O_42_ | 1898.36548 | 1897.357655 | 1897.359875 | -1.17 | 948.18 (M − 2H)^2−^ |
| 143 | CT | Procyanidin dimer monogallate | 13.95 | C_37_H_30_O_16_ | 730.15339 | 729.145565 | 729.146665 | -1.51 | 289.07; 407.08; 577.14 |
| 144 | CT | Procyanidin pentamer tetragallate | 13.96 | C_103_H_78_O_46_ | 2050.37644 | 2049.368615 | 2049.370495 | -0.92 | 1024.18 (M − 2H)^2−^ |
| 145 | CT | Procyanidin hexamer trigallate | 14.09 | C_111_H_86_O_48_ | 2186.42887 | 2185.421045 | 2185.421765 | -0.33 | 1092.21 (M − 2H)^2−^ |
| 146 | CT | Procyanidin haxamer one A-type linkage | 14.16 | C_90_H_72_O_36_ | 1728.38034 | 1727.372515 | 1727.374645 | -1.23 | 863.18 (M − 2H)^2−^ |
| 147 | CT | Procyanidin tetramer one A-type linkage | 14.17 | C_60_H_48_O_24_ | 1152.25356 | 1151.245735 | 1151.247085 | -1.17 | 423,07; 575.12; 863.18 |
| 148 | FN | Chrysoeriol hexoside | 14.18 | C_22_H_22_O_11_ | 462.116215 | 461.10839 | 461.108915 | -1.14 | 298.05; 323.06; 341.04 |
| 149 | LN | Matairesinoside | 14.27 | C_26_H_32_O_11_ | 520.194465 | 519.18664 | 519.187275 | -1.22 | 151.04; 357.13 |
| 150 | CT | Procyanidin tetramer | 14.30 | C_60_H_50_O_24_ | 1154.26921 | 1153.261385 | 1153.261245 | 0.12 | 287.06; 449.09; 575.12; 865.20; 1027.23 |
| 151 | CT | Procyanidin hexamer trigallate | 14.32 | C_111_H_86_O_48_ | 2186.42887 | 2185.421045 | 2185.421765 | -0.33 | 1092.21 (M − 2H)^2−^ |
| 152 | CT | Procyanidin trimer digallate | 14.39 | C_59_H_46_O_26_ | 1170.22774 | 1169.219915 | 1169.221935 | -1.73 | 287.06; 575.12; 881.16; 1083.70 |
| 153 | FO | Quercetin-xyloside | 14.47 | C_20_H_18_O_11_ | 434.084915 | 433.07709 | 433.077545 | -1.05 | 300.03; 301.04 |
| 154 | CT | Procyanidin trimer monogallate | 14.48 | C_52_H_42_O_22_ | 1018.21678 | 1017.208955 | 1017.209485 | -0.52 | 287.06; 729.15; 891.18 |
| 155 | HCA | Dicaffeoylquinic acid | 14.49 | C_25_H_24_O_12_ | 516.12678 | 515.118955 | 515.119325 | -0.72 | 173.04; 179.03; 191.06, 353.09 |
| 156 | CT | Procyanidin tetramer tetragallate | 14.56 | C_88_H_66_O_40_ | 1762.31305 | 1761.305225 | 1761.307755 | -1.44 | 880.15 (M − 2H)^2−^ |
| 157 | FO | Quercetin-malonyl-glucoside | 14.62 | C_24_H_22_O_15_ | 550.095875 | 549.08805 | 549.088805 | -1.38 | 300.03; 301.04 |
| 158 | CT | Procyanidin trimer monogallate | 14.63 | C_52_H_42_O_22_ | 1018.21678 | 1017.208955 | 1017.209675 | -0.71 | 287.06; 729.15; 891.18 |
| 159 | CT | Procyanidin pentamer tetragallate | 14.63 | C_103_H_78_O_46_ | 2050.37644 | 2049.368615 | 2049.370495 | -0.92 | 1024.18 (M − 2H)^2−^ |
| 160 | CT | Procyanidin tetramer trigallate | 14.67 | C_81_H_62_O_36_ | 1610.30209 | 1609.294265 | 1609.296885 | -1.63 | 804.14 (M − 2H)^2−^ |
| 161 | CT | Procyanidin one A-type linkage tetramer digallate | 14.67 | C_74_H_56_O_32_ | 1456.27548 | 1455.267655 | 1455.269305 | -1.13 | 727.13 (M − 2H)^2−^ |
| 162 | FO | Quercetin-acetyl-glucoside | 14.73 | C_23_H_22_O_13_ | 506.106045 | 505.09822 | 505.098801 | -1.15 | 301.04; 463.03 |
| 163 | FN | Luteolin hexoside | 14.76 | C_21_H_20_O_11_ | 448.100565 | 447.09274 | 447.093265 | -1.17 | 227.03; 255.03; 284.03; 285.04 |
| 164 | CT | Procyanidin hexamer tetragallate | 14.79 | C_118_H_90_O_52_ | 2338.43983 | 2337.432005 | 2337.432755 | -0.32 | 1168.21 (M − 2H)^2−^ |
| 165 | CT | Procyanidin hexamer trigallate | 14.97 | C_111_H_86_O_48_ | 2186.42887 | 2185.421045 | 2185.421765 | -0.33 | 1092.21 (M − 2H)^2−^ |
| 166 | CT | Procyanidin tetramer tetragallate | 15.04 | C_88_H_66_O_40_ | 1762.31305 | 1761.305225 | 1761.307755 | -1.44 | 880.15 (M − 2H)^2−^ |
| 167 | CT | Procyanidin trimer trigallate | 15.21 | C_66_H_50_O_30_ | 1322.2387 | 1321.230875 | 1321.232685 | -1.37 | 660.11 (M − 2H)^2−^ |
| 168 | FO | Quercetin-xyloside | 15.25 | C_20_H_18_O_11_ | 434.084915 | 433.07709 | 433.077545 | -1.05 | 300.03; 301.04 |
| 169 | CT | Procyanidin trimer one A-type linkage digallate | 15.29 | C_59_H_44_O_26_ | 1168.21209 | 1167.204265 | 1167.205705 | -1.23 | 287.06; 441.08; 575.12; 727.13; 881.16; 1024.68 |
| 170 | CT | Procyanidin pentamer tetragallate | 15.39 | C_103_H_78_O_46_ | 2050.37644 | 2049.368615 | 2049.370495 | -0.92 | 1024.18 (M − 2H)^2−^ |
| 171 | FN | Luteolin-glucuronide | 15.48 | C_21_H_18_O_12_ | 462.07983 | 461.072005 | 461.072665 | -1.43 | 285.04 |
| 172 | FO | Quercetin-rhamnoside | 15.57 | C_21_H_20_O_11_ | 448.100565 | 447.09274 | 447.093255 | -1.15 | 300.03; 301.04 |
| 173 | FO | Isorhamnetin hexoside | 15.60 | C_22_H_22_O_12_ | 478.11113 | 477.103305 | 477.103735 | -0.90 | 314.04; 315.05 |
| 174 | CT | Procyanidin tetramer trigallate | 15.63 | C_81_H_62_O_36_ | 1610.30209 | 1609.294265 | 1609.296765 | -1.55 | 804.14 (M − 2H)^2−^ |
| 175 | CT | Procyanidin dimer monogallate | 15.70 | C_37_H_30_O_16_ | 730.15339 | 729.145565 | 729.146545 | -1.34 | 289.07; 407.08 |
| 176 | CT | Procyanidin trimer digallate | 15.73 | C_59_H_46_O_26_ | 1170.22774 | 1169.219915 | 1169.221575 | -1.42 | 287.06; 575.12; 729.15; 881.16 |
| 177 | FN | Tricin | 15.90 | C_17_H_14_O_7_ | 330.073955 | 329.06613 | 329.066625 | -1.50 | 243.03; 271.03; 299,02; 314.04 |
| 178 | CT | Procyanidin tetramer one A-type linkage monogallate | 15.92 | C_67_H_52_O_28_ | 1304.26452 | 1303.256695 | 1303.259165 | -1.89 | 651.13 (M − 2H)^2−^ |
| 179 | DHCH | Phloretin hexoside | 15.96 | C_21_H_24_O_10_ | 436.13695 | 435.129125 | 435.129605 | -1.10 | 169.01; 211.02; 271.05; |
| 180 | CT | Procyanidin trimer digallate | 16.11 | C_59_H_46_O_26_ | 1170.22774 | 1169.219915 | 1169.221575 | -1.42 | 287.06; 441.08; 575.12; 729.15; 881.16; 1043.19 |
| 181 | CT | Procyanidin tetramer monogallate | 16.23 | C_67_H_54_O_28_ | 1306.28017 | 1305.272345 | 1305.274185 | -1.41 | 652.13 (M − 2H)^2−^ |
| 182 | HCA | Dicaffeoylquinic acid | 16.23 | C_25_H_24_O_12_ | 516.12678 | 515.118955 | 515.119325 | -0.72 | 173.04; 179.03; 191.06; 353.09 |
| 183 | CT | Procyanidin pentamer monogallate | 16.34 | C_82_H_66_O_34_ | 1594.34356 | 1593.335735 | 1593.338755 | -1.89 | 796.17 (M − 2H)^2−^ |
| 184 | CT | Procyanidin tetramer digallate | 16.37 | C_74_H_58_O_32_ | 1458.29113 | 1457.283305 | 1457.285535 | -1.53 | 728.14 (M − 2H)^2−^ |
| 185 | CT | Procyanidin dimer one A-type linkage | 16.53 | C_30_H_24_O_12_ | 576.12678 | 575.118955 | 575.119625 | -1.16 | 287.06; 394.07 |
| 186 |  | Unknown | 16.65 | C_14_H_16_O_6_ | 280.09469 | 279.086865 | 279.087095 | -0.82 | 149.02; 191.03; 261.08 |
| 187 | CT | Procyanidin tetramer trigallate | 16.90 | C_81_H_62_O_36_ | 1610.30209 | 1609.294265 | 1609.296275 | -1.25 | 804.14 (M − 2H)^2−^ |
| 188 | FN | Luteolin-hexoside | 16.932 | C_21_H_20_O_11_ | 448.100565 | 447.09274 | 447.093265 | -1.17 | 285.04 |
| 189 | CT | Procyanidin trimer trigallate | 16.941 | C_66_H_50_O_30_ | 1322.2387 | 1321.230875 | 1321.232685 | -1.37 | 660.11 (M − 2H)^2−^ |
| 190 | CT | Procyanidin tetramer tetragallate | 16.947 | C_88_H_66_O_40_ | 1762.31305 | 1761.305225 | 1761.307145 | -1.09 | 880,15 (M − 2H)^2−^ |
| 191 | DHCH | Phloretin hexoside | 17.053 | C_21_H_24_O_10_ | 436.13695 | 435.129125 | 435.129655 | -1.22 | 167.03; 273.08 |
| 192 | CT | Procyanidin tetramer monogallate | 17.14 | C_67_H_54_O_28_ | 1306.28017 | 1305.272345 | 1305.274555 | -1.69 | 652.13 (M − 2H)^2−^ |
| 193 | FNN | Naringenin hexoside | 17.16 | C_21_H_22_O_10_ | 434.1213 | 433.113475 | 433.114015 | -1.25 | 270.05; 271.06 |
| 194 | CT | Procyanidin dimer digallate | 17.30 | C_44_H_34_O_20_ | 882.16435 | 881.156525 | 881.157545 | -1.16 | 287.06; 407.08; 559.13; 729.15 |
| 195 | FT | Procyanidin tetramer trigallate | 17.36 | C_81_H_62_O_36_ | 1610.30209 | 1609.294265 | 1609.296275 | -1.25 | 804.14 (M − 2H)^2−^ |
| 196 | FNN | Naringenin hexoside | 18.25 | C_21_H_22_O_10_ | 434.1213 | 433.113475 | 433.113905 | -0.99 | 270.05; 271.06 |
| 197 | CT | Procyanidin trimer trigallate | 18.31 | C_66_H_50_O_30_ | 1322.2387 | 1321.230875 | 1321.233045 | -1.64 | 660.11 (M − 2H)^2−^ |
| 198 | CT | Procyanidin tetramer tetragallate | 18.70 | C_88_H_66_O_40_ | 1762.31305 | 1761.305225 | 1761.307145 | -1.09 | 880.15 (M − 2H)^2−^ |
| 199 |  | Unknown | 19.24 | C_20_H_22_O_7_ | 374.136555 | 373.12873 | 373.129185 | -1.22 | 269.08; 311.09 |
| 200 | NP | Nepodin hexoside | 19.62 | C_19_H_22_O_8_ | 378.13147 | 377.123645 | 377.124055 | -1.09 | 215.07; 216.07 |
| 201 | FO | Quercetin-feruloylhexoside | 19.82 | C_31_H_28_O_15_ | 640.142825 | 639.135 | 639.136175 | -1.84 | 302.04; 463.06 |
| 202 |  | Unknown | 20.21 | C_20_H_22_O_7_ | 374.136555 | 373.12873 | 373.129185 | -1.22 | 269.08; 358.11 |
| 203 | NP | Torachrysone hexoside | 20.79 | C_20_H_24_O_9_ | 408.142035 | 407.13421 | 407.134645 | -1.07 | 230,06; 245,08 |
| 204 | FNN | Liquirtin | 21.05 | C_21_H_22_O_9_ | 418.126385 | 417.11856 | 417.119265 | -1.69 | 254.06; 256.07; 297.08 |
| 205 | AQ | Emodin hexoside | 21.40 | C_21_H_22_O_10_ | 432.10565 | 431.097825 | 431.097995 | -0.39 | 269.05 |
| 206 | FNN | Eriodictyol | 21.43 | C_15_H_12_O_6_ | 288.06339 | 287.055565 | 287.055865 | -1.04 | 135.04; 151.00 |
| 207 | IF | Daidzin | 21.59 | C_15_H_10_O_4_ | 416.110735 | 415.10291 | 415.103515 | -1.46 | 253.05 |
| 208 |  | Unknown | 21.73 | C_17_H_12_O_8_ | 344.05322 | 343.045395 | 343.045865 | -1.376 | 283.03 |
| 209 | FN | Luteolin | 22.00 | C_15_H_10_O_6_ | 286.04774 | 285.039915 | 285.040225 | -1.09 | 133.03; 151.00; 175.00; 190.04 |
| 210 | FO | Quercetin | 22.22 | C_15_H_10_O_7_ | 302.042655 | 301.03483 | 301.035135 | -1.01 | 151.00; 179.00 |
| 211 | NP | Hastatuside B | 22.49 | C_21_H_24_O_9_ | 420.142035 | 419.13421 | 419.134915 | -1.68 | 215.07 |
| 212 | IF | Daidzein | 22.76 | C_15_H_10_O_4_ | 254.05791 | 253.050085 | 253.050065 | 0.08 | 225.06 |
| 213 | AQ | Emodin malonylglucoside | 23.07 | C_24_H_22_O_13_ | 518.106045 | 517.09822 | 517.098755 | -1.03 | 269.05; 473.11 |
| 214 | NP | Nepodin | 23.13 | C_13_H_12_O_3_ | 216.078645 | 215.07082 | 215.070425 | 1.84 | 144.06; 172.05; 173.06 |
| 215 | FO | Isorhamnetin | 23.38 | C_16_H_12_O_7_ | 316.058305 | 315.05048 | 315.051055 | -1.83 | 255.03; 271.02; 300.02 |
| 216 |  | Unknown | 23.46 | C_21_H_22_O_8_ | 402.13147 | 401.123645 | 401.124145 | -1.25 | 281.08 |
| 217 | CM | Isopimpinellin | 23.47 | C_13_H_10_O_5_ | 246.052825 | 245.045 | 245.045065 | -0.27 | 175,04; 189.02; 217.05 |
| 218 | AQ | Emodic acid | 23.56 | C_15_H_8_O_7_ | 300.027005 | 299.01918 | 299.019565 | -1.29 | 227.03; 255.03; 271.02; 299.02 |
| 219 |  | Unknown | 24.22 | C_15_H_12_O_5_ | 272.068475 | 271.06065 | 271.061005 | -1.31 | 143.05; 209.06; 253.05 |
| 220 | AQ | Emodin hexoside | 24.25 | C_21_H_22_O_10_ | 432.10565 | 431.097825 | 431.098275 | -1.04 | 269.05 |
| 221 | FA | Oxo-dihydroxy-octadecenoic acid isomer | 24.26 | C_18_H_32_O_5_ | 328.224975 | 327.21715 | 327.217595 | -1.36 | 171.10; 211.13; 229.14 |
| 222 | IF | Glycitin | 24.37 | C_22_H_22_O_10_ | 446.1213 | 445.113475 | 445.114075 | -1.35 | 240.04; 283.06 |
| 223 | IF | Acetyldaidzin | 24.67 | C_23_H_22_O_10_ | 458.1213 | 457.113475 | 457.114255 | -1.71 | 253.05 |
| 224 | FA | Phloionic acid | 25.07 | C_18_H_34_O_6_ | 346.23554 | 345.227715 | 345.228085 | -1.07 | 247.12; 265.22; 309.21; 327.22 |
| 225 | DHCH | Phloretin | 25.19 | C_15_H_14_O_5_ | 274.084125 | 273.0763 | 273.076725 | -1.56 | 13.04; 151.00; 167.03 |
| 226 | AQ | Endocrocin | 25.55 | C_16_H_10_O_7_ | 314.042655 | 313.03483 | 313.035275 | -1.42 | 225.06; 269.05 |
| 227 | FNN | Naringenin | 25.66 | C_15_H_12_O_5_ | 272.068475 | 271.06065 | 271.061005 | -1.31 | 119.05; 151.00; 177.02 |
| 228 | FNN | Hesperetin | 26.11 | C_16_H_14_O_6_ | 302.07904 | 301.071215 | 301.071625 | -1.36 | 134.04; 151.00 |
| 229 | FA | Pinellic acid | 26.27 | C_18_H_34_O_5_ | 330.240625 | 329.2328 | 329.233175 | -1.14 | 171.10; 183.14; 193.12; 211.13 |
| 230 | FN | Hispidulin | 26.66 | C_16_H_12_O_6_ | 300.06339 | 299.055565 | 299.055935 | -1.24 | 256.04; 284.03 |
| 231 |  | Unknown | 26.79 | C_15_H_10_O_6_ | 286.04774 | 285.039915 | 285.040225 | -1.09 | 133.03; 151.00; 181.90; 229.05 |
| 232 | AQ | Hydroxyemodin | 27.29 | C_15_H_10_O_6_ | 286.04774 | 285.039915 | 285.040225 | -1.09 | 211.04; 241.05; 257.05; 268.04 |
| 233 | FO | Dimethylquercetin | 28.19 | C_17_H_14_O_7_ | 330.073955 | 329.06613 | 329.066655 | -1.59 | 243.03; 271.03; 285.04; 299.02 |
| 234 | FN | Apigenin-sulfate | 28.39 | C_15_H_10_O_8_S | 350.009642 | 349.001817 | 349.002075 | -0.74 | 269.05 |
| 235 | FN | Apigenin | 28.39 | C_15_H_10_O_5_ | 270.052825 | 269.045 | 269.044705 | 1.09 | 225.06; 241.05 |
| 236 | AQ | Rhein methylester | 29.15 | C_16_H_10_O_6_ | 298.04774 | 297.039915 | 297.040255 | -1.14 | 225.06; 253.05 |
| 237 | AQ | Emodin | 31.53 | C_15_H_10_O_5_ | 270.052825 | 269.045 | 269.045255 | -0.95 | 225.06; 241.05 |
| 238 |  | Unknown | 33.90 |  |  |  | 1017.610045 |  | 671.40; 973.62 |
| 239 | FA | 3-Hydroxyhexadecanoic acid | 34.86 | C_16_H_32_O_3_ | 272.235145 | 271.22732 | 271.227635 | -1.16 | 225.22 |
| 240 | FA | 1-Heptadecanoyl-rac-glycerol | 35.27 | C_20_H_40_O_4_ | 344.29266 | 343.284835 | 343.285155 | -0.93 | 72.99 |
| 241 |  | Unknown | 35.34 | C_41_H_84_O_3_S_2_ | 688.586189 | 687.578364 | 687.578615 | -0.37 | 372.34; 641.57 |
| 242 | TT | Ursolic acid | 37.08 | C_30_H_48_O_3_ | 456.360345 | 455.35252 | 455.352875 | -0.78 | 455.35 |
| 243 | TP | Tocopherol acetate | 37.66 | C_31_H_52_O_3_ | 472.391645 | 471.38382 | 471.384215 | -0.84 | 471.38 |
| 244 | TP | Tocopherol acetate | 38.37 | C_31_H_52_O_3_ | 472.391645 | 471.38382 | 471.384215 | -0.84 | 145.03 |

*HBA- hydroxybenzoic acids and derivatives; HCA - hydroxycinnamic acids and derivatives; F-3-ol - flavan-3-ols; HT- hydrolysable tannins and derivatives; CT - condensed tannins and derivatives; IF - isoflavones; FNN - flavanones, FO - flavonols; FN - flavones; LN - lignans; DHCH - dihydrochalcones and derivatives; FNO - flavanonols; AQ -anthraguinones and derivatives; CM - coumarins and derivatives; NP - naphthols; FA - fatty acids; TT - triterpenoids; TP - tocopherols

**Supplementary table 2. Major identified compounds in *R. obtusifolius* extract with possible antioxidant, anticancer, and anti-inflammatory properties**

| **Compound Group** | **Tentative identification** | **Post-column derivatization with ABTS (+/-)*** | **Biological (anticancer, antioxidant, anti-inflammatory) activities** |
| --- | --- | --- | --- |
| flavan-3-ols | Catechin | + | Antioxidant, anticancer ^1,2^ |
|  | Epicatechin | + | Antioxidant, anticancer ^1,2^ |
|  | (epi)Catechin monogallate | + | Antioxidant ^1,2^ |
| hydroxybenzoic acids and derivatives | Glucogallic acid isomer | - | N/A |
|  | Hamamelitannin | - | Antioxidant, anticancer ^3,4^ |
|  | Gallic acid | + | Anticancer, antioxidant, ^1,2^ |
|  | Protocatechuic acid | + | Antioxidant ^5^ |
|  | Hydroxybenzoic acid | – | аntioxidant ^6^ |
| condensed tannins and derivatives | Procyanidin dimer | + | аntioxidant, anticancer ^7,8^  N/A |
|  | Procyanidin tetramer one A-type linkage | + |  |
|  | Procyanidin trimer one A-type linkage | + |  |
|  | Procyanidin tetramer | + |  |
|  | Procyanidin dimer monogallate | + |  |
|  | Procyanidin trimer digallate | + |  |
|  | Procyanidin dimer digallate | + |  |
|  | Procyanidin trimer trigallate | + |  |
| flavonols | Quercetin-diglucoside | - | Antioxidant, anticancer ^9^ |
|  | Isorhamnetin hexoside | - | N/A |
|  | Quercetin-hexoside | - | N/A |
|  | Quercetin-xyloside | - | Antioxidant ^10^ |
|  | Quercetin | - | Antioxidant, anti-inflammatory, and anticancer ^1,11,12^ |
| lignans | Lariciresinol hexoside | - | N/A |
| naphthols | Nepodin hexoside | - | N/A |
|  | Torachrysone hexoside | - | N/A |
| flavanones | Eriodictyol | - | antioxidant, anti-inflammatory, and anticancer ^1^ |
| flavones | Luteolin | - | antioxidant, anticancer, and anti-inflammatory (Ganai et al., 2021; Swamy, 2020) |
|  | Apigenin-sulfate | - | N/A |
| anthraquinones and derivatives | Endocrine | - | N/A |
|  | Hydroxyemodin | - | antioxidant and anti-inflammatory (Bhatarrai et al., 2021) |
|  | Rhein methyl ester | - | N/A |
|  | Emodin | - | anticancer (Swamy, 2020; Wagh et al., 2020) |

* Antioxidant profiling was done by HPLC coupled post-column derivatization with ABTS reagent. “+” – presence of antioxidant activity, “-” – absence of antioxidant activity, “N/A” – not applicable.


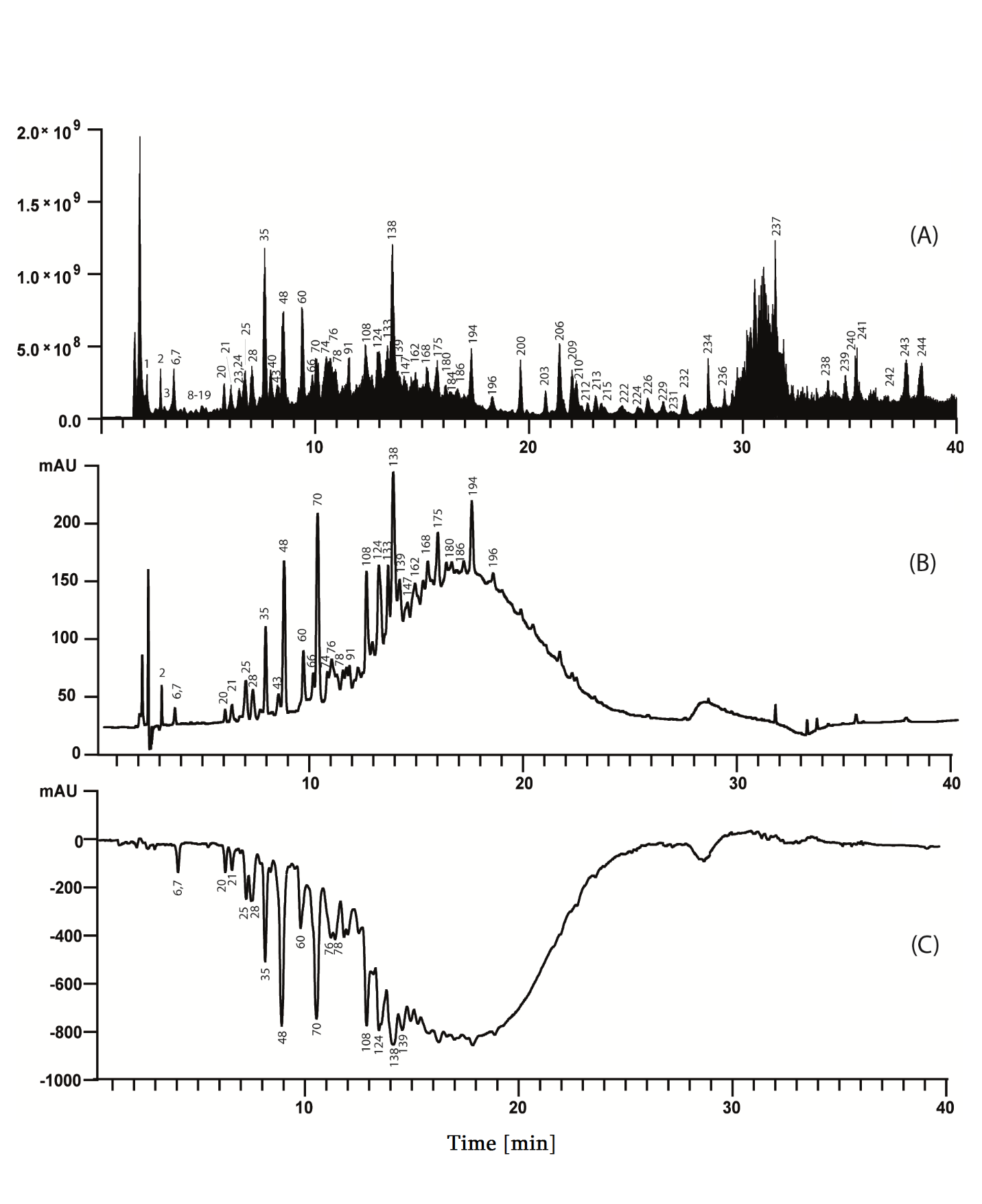


**Figure. Chemical analysis of RO seed extract composition: (A) - HPLC-MS, (B) - HPLC-DAD monitored at 270 nm, (C) – antioxidant profiling by post-column derivatization with ABTS radical monitored at 734 nm.**

## Methods

## 1. Plant material characterization (LC-Q-Orbitrap HRMS analysis)

The *R. obtusifolius* extract was investigated using a Dionex Ultimate 3000 UHPLC system (Thermo Scientific TM, Dionex, San Jose, CA, USA) equipped with a SynergyTM Hydro-RP A (150 x 4.5 mm, 4 µm, Phenomenex) column that was held at a temperature of 30°C. The injection volume was 1 μL. The mobile phase was constituted by water (solvent A) and acetonitrile (solvent B), and both were acidified with formic acid (0.1% v/v). A gradient elution was performed at a flow rate of 800 μL/min, according to the following gradient program: 0 min, 10% B; 25 min 40% B; 30 min 100% B; 35 min 100%, and finally, the initial conditions were held for 8 min as a re-equilibration step.

The chromatographic system was coupled to a Q Exactive^TM^ Focus quadrupole-Orbitrap mass spectrometer (Thermo Fisher Scientific, Bremen, Germany) with a heated electrospray ionization source (HESI II). Detection was performed using a Q-Exactive mass spectrometer. The HESI parameters in negative polarity were as follows: sheath gas flow rate, 35; auxiliary gas flow rate, 15; sweep gas flow rate, 3; spray voltage, 2.5 kV; capillary temperature, 350°C; S-lens RF level, 50; heater temperature, 300°C. The parameters of the full-scan analysis were as follows: resolution, 70,000; AGC target, 1e6; max IT, auto; scan range, 120-1200. The parameters of the data-dependent MS2 were as follows: resolution, 17,500; isolation window, 3.0 m/z; normalized collision energy, 30; AGC target, 1e6; max IT, auto.

The raw data from HRMS were elaborated with Compound Discoverer (v. 2.1, Thermo, Waltham, USA), which facilitated the peak recognition, retention times arrangement, profile alignment, and isotope pattern. Major metabolite identification was based on the accurate mass and mass fragmentation pattern spectra against the MS-MS spectra of the compounds available in a customized database of different classes of phytochemicals created based on data from the literature that was implemented in the software. The raw data from three experimental replicates and a blank sample were processed using the workflow described by Kusznierewicz et al. (Kusznierewicz et al., 2021).

## Antioxidant profiling using post-column derivatization with ABTS

The *R. obtusifolius* antioxidant profiling was performed using the HPLC-DAD system (Agilent Technologies, Wilmington, DE, USA), which was connected to a Pinnacle PCX Derivatization Instrument (Pickering Laboratories Inc., Mountain View, California, USA) and a UV–Vis’s detector (Agilent Technologies, Wilmington, DE, USA). The chromatographic separation conditions were the same as the LC-HRMS analysis. The post-column derivatization with the ABTS reagent was carried out according to Kusznierewicz et al. ^17^ with a slight modification. A stream of methanolic ABTS solution (1 mM) was introduced to the stream of eluate at a flow rate of 0.1 mL/min and then was directed to the reaction loop (1 mL, 130°C). The antioxidant profiles were recorded by a UV-Vis detector at 734 nm.

**References**

1. Swamy, M. K. *Plant-Derived Bioactives*. (Springer Singapore, 2020).

2. Baranowska, M. *et al.* The relationship between standard reduction potentials of catechins and biological activities involved in redox control. *Redox Biol* **17**, 355–366 (2018).

3. Antonsen, S., Østby, R. B. & Stenstrøm, Y. *Naturally Occurring Cyclobutanes: Their Biological Significance and Synthesis*. *Studies in Natural Products Chemistry* vol. 57 (2018).

4. Masaki, H., Atsumi, T. & Sakurai, H. Peroxyl radical scavenging activities of hamamelitannin in chemical and biological systems. *Free Radic Res* **22**, 419–430 (1995).

5. Keskin, C., Aktepe, N., Yukselten, Y., Asuman & Boga, M. *In-vitro*Antioxidant, Cytotoxic, Cholinesterase Inhibitory Activities\nand Anti-Genotoxic Effects of *Hypericum retusum* Aucher Flowers, Fruits\nand Seeds Methanol Extracts in Human Mononuclear Leukocytes. *Iranian Journal of Pharmaceutical Research* **16**, 210–220 (2017).

6. Farhoosh, R., Johnny, S., Asnaashari, M., Molaahmadibahraseman, N. & Sharif, A. Structure – antioxidant activity relationships of flavonoids isolated from different plant species Related papers. *Food Chem* **194**, 128–134 (2016).

7. Kumar, S. & Pandey, A. K. Chemistry and Biological Activities of Flavonoids : An Overview. *The Scientific World Journal* 1–16 (2013).

8. Li, W. *et al.* Chemical characterization of procyanidins from Spatholobus suberectus and their antioxidative and anticancer activities. *J Funct Foods* **12**, 468–477 (2015).

9. Taiwo, B.J., Popoola, T. D., van Heerden, F. R. & Fatokun, A. A. LJMU Research Online ｍ. *Chem Biodivers* **18**, e2000670 (2021).

10. Yan, X., Murphy, B. T., Hammond, G. B., Vinson, J. A. & Neto, C. C. Antioxidant activities and antitumor screening of extracts from Cranberry fruit (Vaccinium macrocarpon). *J Agric Food Chem* **50**, 5844–5849 (2002).

11. Nour, V., Trandafir, I. & Cosmulescu, S. Antioxidant capacity, phenolic compounds and minerals content of blackcurrant (ribes nigrum L.) leaves as influenced by harvesting date and extraction method. *Ind Crops Prod* **53**, 133–139 (2014).

12. Kumar, S. & Pandey, A. K. Chemistry and Biological Activities of Flavonoids : An Overview. *The Scientific World Journal* 1–16 (2013).

13. Ganai, S. A. *et al.* Anticancer activity of the plant flavonoid luteolin against preclinical models of various cancers and insights on different signalling mechanisms modulated. *Phytotherapy Research* **35**, 3509–3532 (2021).

14. Bhatarrai, G., Choi, J., Seong, S. H. & Nam, T. Activities of Alaternin ( = 7-Hydroxyemodin ). **27**, 28–35 (2021).

15. Wagh, N. S., Pai, S. R. & Sonkamble, V. V. Phytochemicals in the Prevention and Cure of Cancers. *Plant-derived Bioactives* 351–373 (2020) doi:10.1007/978-981-15-2361-8_16.

16. Kusznierewicz, B., Mróz, M., Koss-Mikołajczyk, I. & Namieśnik, J. Comparative evaluation of different methods for determining phytochemicals and antioxidant activity in products containing betalains – Verification of beetroot samples. *Food Chem* **362**, 130132 (2021).

17. Kusznierewicz, B., Piasek, A., Bartoszek, A. & Namiesnik, J. Application of a commercially available derivatization instrument and commonly used reagents to HPLC on-line determination of antioxidants. *Journal of Food Composition and Analysis* **24**, 1073–1080 (2011).
